# Functional Evolution of *cis*-Regulatory Modules at a Homeotic Gene in *Drosophila*

**DOI:** 10.1371/journal.pgen.1000709

**Published:** 2009-11-06

**Authors:** Margaret C. W. Ho, Holly Johnsen, Sara E. Goetz, Benjamin J. Schiller, Esther Bae, Diana A. Tran, Andrey S. Shur, John M. Allen, Christoph Rau, Welcome Bender, William W. Fisher, Susan E. Celniker, Robert A. Drewell

**Affiliations:** 1 Biology Department, Harvey Mudd College, Claremont, California, United States of America; 2 College of Osteopathic Medicine of the Pacific, Western University of Health Sciences, Pomona, California, United States of America; 3 Department of Biological Chemistry and Molecular Pharmacology, Harvard Medical School, Boston, Massachusetts, United States of America; 4 Berkeley Drosophila Genome Project, Lawrence Berkeley National Laboratory, Berkeley, California, United States of America; Princeton University, Howard Hughes Medical Institute, United States of America

## Abstract

It is a long-held belief in evolutionary biology that the rate of molecular evolution for a given DNA sequence is inversely related to the level of functional constraint. This belief holds true for the protein-coding homeotic (Hox) genes originally discovered in *Drosophila melanogaster*. Expression of the Hox genes in *Drosophila* embryos is essential for body patterning and is controlled by an extensive array of *cis*-regulatory modules (CRMs). How the regulatory modules functionally evolve in different species is not clear. A comparison of the CRMs for the *Abdominal-B* gene from different *Drosophila* species reveals relatively low levels of overall sequence conservation. However, embryonic enhancer CRMs from other *Drosophila* species direct transgenic reporter gene expression in the same spatial and temporal patterns during development as their *D. melanogaster* orthologs. Bioinformatic analysis reveals the presence of short conserved sequences within defined CRMs, representing gap and pair-rule transcription factor binding sites. One predicted binding site for the gap transcription factor KRUPPEL in the IAB5 CRM was found to be altered in *Superabdominal* (*Sab*) mutations. In *Sab* mutant flies, the third abdominal segment is transformed into a copy of the fifth abdominal segment. A model for KRUPPEL-mediated repression at this binding site is presented. These findings challenge our current understanding of the relationship between sequence evolution at the molecular level and functional activity of a CRM. While the overall sequence conservation at *Drosophila* CRMs is not distinctive from neighboring genomic regions, functionally critical transcription factor binding sites within embryonic enhancer CRMs are highly conserved. These results have implications for understanding mechanisms of gene expression during embryonic development, enhancer function, and the molecular evolution of eukaryotic regulatory modules.

## Author Summary

The fertilized animal embryo is a mass of uniform cells that becomes a complex, segmented, and highly organized structure of differentiated cells through the process of development. This vital process is controlled by networks of developmental genes interacting with each other on the molecular level. Because these genes are crucial for animal development, they are conserved both in function and at the DNA sequence level in related species. We have examined critical DNA sequence modules which regulate genes that pattern the early embryo in different species of the fruit fly. We found that despite rapid evolution of the DNA sequences, the regulatory sequences from one fruit fly species are able to operate when tested in another fruit fly species. Further analysis reveals that there are sequences within these regulatory DNA modules which are conserved across different species and which are critical for regulatory function. These conserved sequences represent critical binding sites for protein transcription factors. These findings have important implications for our understanding of gene regulation during development and evolution across diverse animal species ranging from the fruit fly to humans.

## Introduction

**Figure 1:**
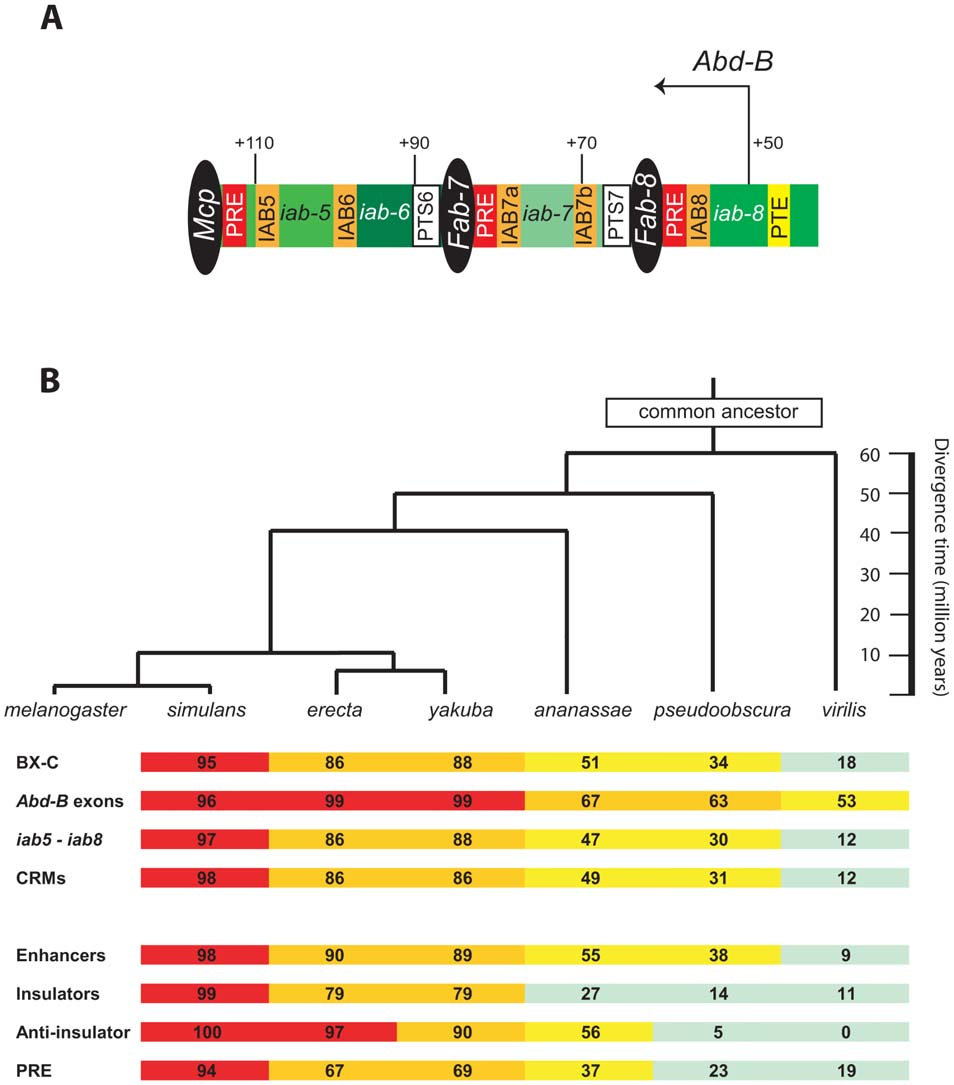
Molecular organization and sequence conservation of the 3′ regulatory regions for the *Abdominal-B* gene in *Drosophila melanogaster*. (A) An extensive array of 3′ *cis*-regulatory modules directs the embryonic expression of the *Abd-B* gene. The *Abd-B* transcription start site is indicated by leftward arrow. The *cis*-regulatory *iab* regions (*iab5–8*) are indicated as shaded rectangles and the characterized enhancers in the individual *iab* regions IAB5, IAB6, IAB7a, IAB7b, and IAB8 are specified with orange rectangles. The positions of the Fab-7, Fab-8, and Mcp insulators, which functionally separate the *iab* regions, are indicated as black ellipses. The promoter targeting sequence (PTS) modules (white rectangles), Polycomb response elements (PREs) (red rectangles) and promoter tethering element (PTE) (yellow rectangle) are also shown. Numbers above the line refer to kilobase positions in DNA sequence accession number: U31961. (B) A consensus tree illustrating evolutionary relationships among *Drosophila* species and sequence conservation at the *Abd-B* gene. Tree indicates evolutionary relationships between *Drosophila* species [67]. Level of conservation between sequences from *D. melanogaster* and six other *Drosophila* species is indicated by color code: >90% red, 60–90% orange, 30–60% yellow, <30% green (calculation for conservation is detailed in Materials and Methods). The sequences listed are the entire bithorax complex (BX-C), exons from the *Abd-B* gene (*Abd-B* exons), the complete 3′ chromosomal region that directs *Abd-B* gene expression during embryonic development (*iab5–iab8*), and the defined CRMs from the *iab5–8* regions (CRMs). The CRMs are further sub-divided to show the level of conservation among the enhancer, insulator, anti-insulator and PRE modules and are described in more detail in Table S1.

The *Drosophila* bithorax complex (BX-C) is over 300 kb in size [1], but contains only three homeotic (Hox) genes, *Ultrabithorax* (*Ubx*), *abdominal-A* (*abd-A*), and *Abdominal-B* (*Abd-B*) [2]. These genes control the identity of ten parasegments (PS5-14) in the posterior thorax and abdomen of the developing fly and are important in the evolution of animal morphology [3]. Extensive genomic regions between the Hox genes in the BX-C, called *infraabdominal* (*iab*) regions, harbor distinct non-genic DNA sequences, called *cis*-regulatory modules (CRMs), which regulate the neighboring Hox genes (Figure 1A) (for recent comprehensive reviews see [4],[5]). One type of CRM, the embryonic enhancer, acts in response to gap and pair-rule factors to initiate specific patterns of transcription for the Hox genes during early embryonic development. Other classes of CRMs include insulators, which act as boundary elements to prevent cross-talk between adjacent *iab* regions [6],[7], and Trithorax and Polycomb response elements, which function to maintain patterns of Hox gene expression or silencing in later developmental stages via chromatin-mediated effects [8],[9].

The BX-C Hox gene *Abd-B* specifies the developmental identity of the 10^th^ to 14^th^ parasegments (abdominal segments 5–9) during *Drosophila melanogaster* development [10]. The *iab-5* to *iab-8* genomic regions each harbor at least one embryonic enhancer CRM which is responsible for driving *Abd-B* expression in specific segments (Figure 1A) [4],[11]. The IAB5 enhancer CRM in the *iab-5* genomic region is capable of driving *Abd-B* expression in the presumptive fifth, seventh, and ninth abdominal segments of *Drosophila melanogaster* [12]. Similarly, the IAB8 enhancer CRM in the *iab-8* region is responsible for driving *Abd-B* expression in the presumptive eighth abdominal segment [13],[14]. Enhancer CRMs usually contain a high number of transcription factor binding sites (TFBSs), strongly indicating that regulation of gene expression by these CRMs is controlled by the binding of specific transcription factors (TFs) [15],[16]. Previous work on the IAB5 enhancer CRM identified several TFs that directly regulate IAB5 activity. IAB5 is thought to mediate transcriptional activation of *Abd-B* by the binding of the pair-rule factor FUSHI-TARAZU (FTZ) [17], which is expressed in seven stripes in the developing embryo. There are currently three reported gap transcriptional repressors known to bind at the IAB5 CRM; KRUPPEL (KR), KNIRPS (KNI) and HUNCHBACK (HB) [17]. KR has been shown to set the anterior boundary for IAB5 activation in the embryo. KNI is thought to be a weak repressor, while the role for HB remains unclear, although previous studies suggest it may act as a direct repressor [17].

The high level of conservation of the homeodomain-coding sequences for the Hox proteins was essential to their discovery in species as diverse as fish, frogs and humans [18]. However, equivalent sequence knowledge does not exist for the evolution of the extensive array of CRMs that are critical for the control of Hox gene expression patterns. Early pioneering research on the evolution of sequence and functional activity at CRMs in *Drosophila* has focused on the *eve stripe 2* enhancer (S2E). In particular, Ludwig and colleagues discovered that the S2Es in *D. yakuba*, *D. erecta* and *D. pseudoobscura*, identified by sequence alignment to the *D. melanogaster* S2E, are able to drive reporter gene expression in transgenic *D. melanogaster* embryos in a comparable spatio-temporal pattern to the endogenous *D. melanogaster* S2E [19]. This evolutionary analysis was recently extended by the Eisen lab to the more evolutionarily divergent scavenger fly (Sepsid) species. The eve stripe 2, stripe 3+7, stripe 4+6 and muscle-heart enhancers from Sepsid species (*S. cynipsea*, *T. putris*, and *T. superba*) are all able to drive reporter gene expression in transgenic *D. melanogaster* in a spatio-temporal pattern comparable to their *D. melanogaster* CRM orthologs [20]. The conservation of the functional activity of these enhancers paradoxically contrasts with the relative lack of overall sequence conservation of the S2E enhancer within *Drosophila* and the more pronounced rearrangement of sequences at the *eve* genomic regulatory region in Sepsid species relative to *Drosophila*. Despite these and other recent advances deciphering other regulatory sequences [21]–[24], there remain many challenges in identifying *Drosophila cis*-regulatory sequences through the application of bioinformatic comparative sequence analysis. In large genomes such as that of vertebrates, high level sequence conservation of a non-protein coding genomic region compared to surrounding genomic regions is often indicative of potential *cis*-regulatory activity [25]–[32]. However, these types of comparative studies have been less successful in small-genome invertebrates such as *Drosophila melanogaster* and *Caenorhabditis elegans* [33],[34].

To address these issues, we compared the sequence conservation at many of the previously identified CRMs for the *Abd-B* gene in the *Drosophila melanogaster* BX-C (Figure 1A). These analyses were made possible by the recent sequencing of twelve *Drosophila* genomes [35]. In this study we analyzed BX-C sequences from seven species spanning approximately 60 million years of evolutionary time: *D. melanogaster*, *D. simulans*, *D. erecta*, *D. yakuba*, *D.ananassae*, *D. pseudoobscura* and *D. virilis* (Figure 1B) [36]. Our experiments demonstrate that despite a distinctive lack of sequence conservation when compared to neighboring genomic regions, the experimentally well-defined IAB5 and IAB8 enhancer CRMs are functionally conserved across the *Drosophila* genus. While overall levels of sequence conservation may not necessarily correlate with functional conservation, sequence homology to known functional CRMs in *D. melanogaster* may assist with the identification of functional CRM orthologs in the other *Drosophila* species. In our quest to further understand the evolution of CRM function at the molecular level, we also developed a more stringent bioinformatic approach to identify highly conserved TFBSs critical for the functional activity of enhancers. It will be of great interest to apply these bioinformatic analyses to the molecular dissection of enhancer function and to identify additional CRMs in the *Drosophila* genome.

## Results

### Evolution of regulatory sequences at the bithorax complex

Bioinformatic analysis of DNA sequence reveals that for the BX-C as a whole and the 3′ control regions of the *Abd-B* gene (*iab5–iab8*), there is a strong correlation between the species divergence time and the level of sequence conservation (Figure 1B and Table S1). In agreement with the biological paradigm that functional regions in the genomes of closely related species are subject to evolutionary constraint, the *Abd-B* exons exhibit a significantly higher level of sequence conservation than the neighboring sequences of the BX-C across all seven *Drosophila* species (Figure 1B, *Abd-B* exons). In contrast, the specific functional CRMs identified in the BX-C do not follow this pattern, but are comparably conserved to the neighboring genomic sequences in all the species analyzed (Figure 1B, CRMs and *iab5–iab8*). Detailed analysis of the sequence conservation and genomic coordinates of DNA regions at the *D. melanogaster* BX-C are shown in Table S1. The trend of a relative lack of sequence conservation is found within each class of CRMs, including enhancers, insulators, anti-insulators and Polycomb-response elements, suggesting that sequences are evolving rapidly at all types of CRMs in the BX-C. The non-protein coding regions of the BX-C are only slightly more conserved across the *Drosophila* genus than the neighboring upstream genomic region of equal size from outside of the BX-C on chromosome 3R and are comparable in level of conservation to the considerably more compact (∼18kb) *eve* gene and associated genomic regulatory regions (Table S1).

### Expression pattern of *Abd-B* is conserved in *Drosophila* species

The lack of sequence conservation of the *iab* regulatory regions and associated CRMs compared to neighboring protein-coding sequences led us to investigate whether the spatio-temporal expression pattern of *Abd-B* in other *Drosophila* species is different from that in *D. melanogaster*. *In situ* hybridization (ISH) with probes against *Abd-B* in embryos collected from the different *Drosophila* species revealed that the expression pattern is conserved in all species at early stages of development (Figure S1) and is localized to abdominal segments 5–9 in late stage embryos (data not shown). This result indicates that the regulation of *Abd-B* gene expression in the embryo may be evolutionarily conserved.

### Sequence conservation at the IAB5 and IAB8 enhancer CRMs

**Figure 2:**
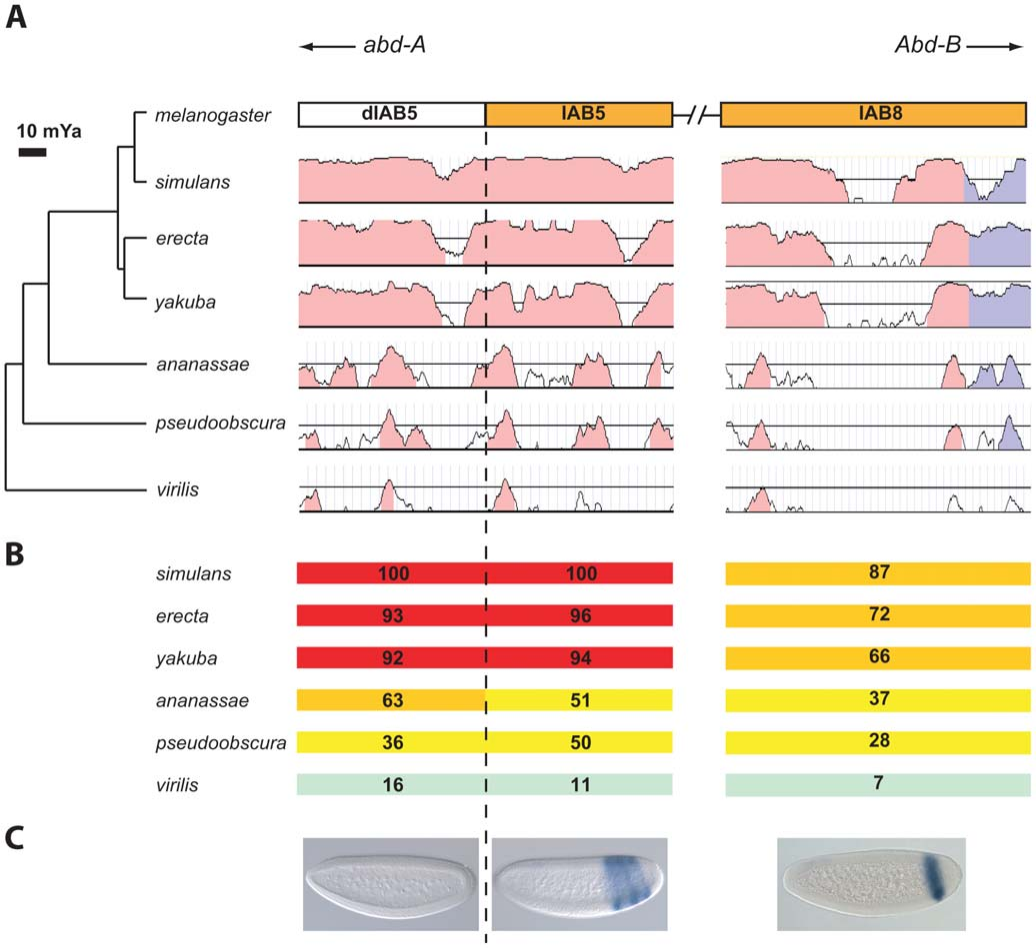
Spatial variation of conservation levels at the bithorax complex *cis*-regulatory modules. (A) VISTA plot of IAB5, dIAB5 and IAB8 genomic regions. The genomic location of the IAB5, dIAB5 and IAB8 regions relative to the neighboring Hox genes (*abd-A* and *Abd-B*) is indicated. Figures of sequence conservation level were generated by VISTA using default parameters. Conserved non-coding sequences (>70% sequence identity over a window of 100 bp) are indicated in pink. Conserved sequence in a region annotated as protein-coding is shown in blue. However, the identified CG10349 sequence located in the IAB8 region currently has no known or predicted function. (B) Level of sequence conservation. Level of conservation between sequences from *D. melanogaster* and six other *Drosophila* species is indicated by color code: >90% red, 60–90% orange, 30–60% yellow, <30% green (calculation for conservation is detailed in Materials and Methods). (C) Enhancer regulatory activity in *Drosophila* embryos. The *D. melanogaster* dIAB5 genomic region (left) does not exhibit enhancer activity in *D. melanogaster* embryos in a transgenic assay (described in Materials and Methods). In contrast, the IAB5 (center) and IAB8 (right) *D. melanogaster* genomic regions drive *lacZ* reporter gene expression in the presumptive fifth, seventh and ninth abdominal segments or the presumptive eighth abdominal segment, respectively, in *D. melanogaster* embryos.

Similar to the other BX-C CRMs, the sequences at the IAB5 and IAB8 enhancer CRMs are no more conserved than neighboring regions of DNA. The 1kb IAB5 [12] and 1.6kb IAB8 [14] enhancers are well-defined regions discovered in transgenic studies. Comparison of IAB5 to the neighboring downstream genomic region of equal length (dIAB5) reveals that the two regions do not demonstrate significant differences in levels of sequence conservation (Figure 2A) and both regions have progressively diminishing levels of sequence conservation in more distantly related *Drosophila* species (Figure 2B). Therefore, the IAB5 CRM appears no more highly conserved than an equal-sized neighboring region of DNA. To compare the functional activity of IAB5 and dIAB5 regions from *D. melanogaster*, they were each tested in transgenic reporter gene assays. In contrast to the IAB5 region, dIAB5 is unable to activate reporter gene expression during any stage of embryonic development (Figure 2C), although this does not preclude the dIAB5 region from other potential functional activities. The sequence conservation of the IAB8 enhancer CRM also rapidly decreases in species more distantly related to *D. melanogaster*. IAB8 exhibits significantly lower levels of sequence conservation across the *Drosophila* genus when compared to the conservation of the IAB5 enhancer (Figure 2B). Indeed, the IAB8 enhancer exhibits the lowest levels of sequence conservation of the known enhancers of the BX-C across the *Drosophila* genus (Table S1).

### Functional equivalence of CRMs from the BX-C in *Drosophila* species

**Figure 3:**
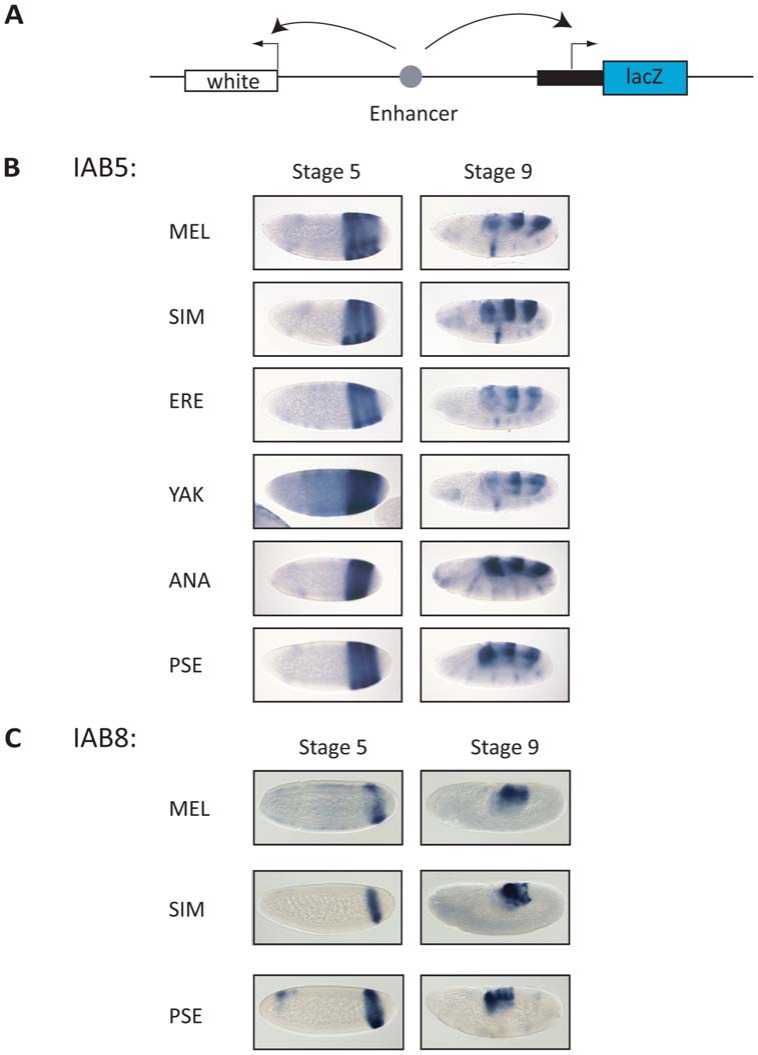
Functional conservation of IAB5 and IAB8 orthologs despite a lack of sequence conservation. (A) *P* element construct for functional assays. A heterologous IAB5 or IAB8 region (gray circle) is inserted between the *white* and *eve*-*lacZ* reporter genes on the *P* element reporter construct to test for conserved functional enhancer activity in transgenic *D. melanogaster* embryos. (B) Transgenic embryos carrying IAB5 orthologs show an IAB5-like *lacZ* expression pattern. The *D. melanogaster* IAB5 CRM (MEL) and orthologs of IAB5, identified by sequence alignment in *D. simulans* (SIM), *D. erecta* (ERE), *D. yakuba* (YAK), *D. ananassae* (ANA), and *D. pseudoobscura* (PSE) drive expression of the reporter gene *lacZ* in the characteristic IAB5 pattern in the presumptive fifth, seventh and ninth abdominal segments of stage 5 and stage 9 *D. melanogaster* embryos. The pattern of *white* expression in embryos carrying these transgenes is identical (data not shown). (C) Transgenic embryos carrying IAB8 orthologs show an IAB8-like *lacZ* expression pattern. The *D. melanogaster* IAB8 CRM and orthologs of IAB8, identified by sequence alignment in *D. simulans* (SIM) and *D. pseudoobscura* (PSE), drive expression of the reporter genes *lacZ* in the characteristic IAB8 pattern in the presumptive eighth abdominal segment of stage 5 and stage 9 *D. melanogaster* embryos. The pattern of *white* expression in embryos carrying these transgenes is identical (data not shown).

The striking lack of underlying sequence conservation demonstrated by the BX-C CRMs suggests that they are evolving rapidly in *Drosophila* species. This prompts the intriguing question of whether the functional activity of a CRM can be conserved in the absence of overall sequence conservation. In order to test this question, we generated transgenic *D. melanogaster* harboring a reporter construct with the orthologous IAB5 or IAB8 sequences from different *Drosophila* species (Figure 3A). Despite the lack of sequence conservation across the *Drosophila* genus, orthologous IAB5 regions, identified by simple sequence alignment using default VISTA values [37] (see Materials and Methods), from each of the six species tested (*D. melanogaster*, *D. simulans*, *D. erecta*, *D. yakuba*, *D.ananassae*, and *D. pseudoobscura*) were able to drive *lacZ* (Figure 3B) and *white* (not shown) reporter gene expression in the fifth, seventh, and ninth abdominal segments. These patterns are evident in both stage 5 and stage 9 embryos and are consistent with the known pattern of IAB5 activity [12],[38]. Despite a more pronounced lack of underlying sequence conservation orthologous IAB8 regions, identified by simple sequence alignment in each of the three species tested (*D. melanogaster*, *D. simulans*, and *D. pseudoobscura*) were also able to drive *lacZ* (Figure 3B) and *white* (not shown) reporter gene expression in a conserved pattern in the eighth abdominal segment of *D. melanogaster* embryos at stage 5 and stage 9 in transgenic assays (Figure 3C) [14]. Note that the additional staining that appears in the anterior region in *Drosophila* embryos when using the *lacZ* ISH probe and is not specific to transgenes carrying the IAB5 or IAB8 enhancers. This ectopic staining anterior staining, which corresponds to thoracic segment T1 in stage 9 embryos (see embryos in Figure 3B), has been documented in the literature as background staining [12],[14] that occurs when using the *lacZ* ISH probe.

### Computational approaches to predict TFBS sequences within CRMs

**Figure 4:**
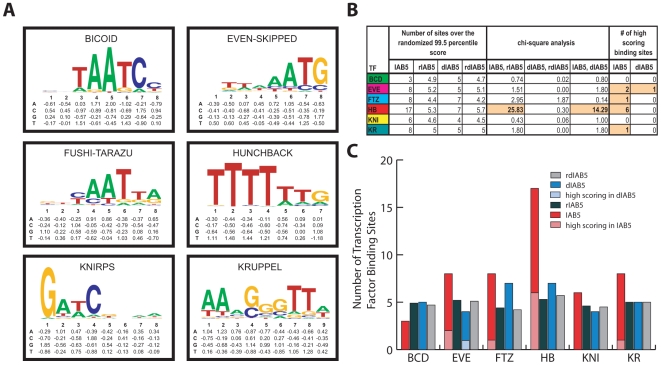
Bioinformatic identification of high-scoring TFBSs in the IAB5 enhancer CRM. (A) TFBS consensus sequences and Position Weight Matrices. TF consensus binding site sequences for BICOID (BCD), EVEN-SKIPPED (EVE), FUSHI-TARAZU (FTZ), HUNCHBACK(HB), KNIRPS (KNI) and KRUPPEL (KR) are shown above the PWM generated from experimentally-verified TF binding sites compiled using databases from the Eisen [39], Siggia [40] and Desplan [41] laboratories in combination with the Transfac public database [42] and additional literature searches (see Materials and Methods and Dataset S1). The height of each of the nucleotide bases reflects the relative likelihood of their presence at that position in the TFBS. (B) Identification of high-scoring TFBSs in IAB5. Rows show each of the TFs; BCD, EVE, FTZ, HB, KNI, and KR. Columns show the number of TFBSs found in IAB5, randomized IAB5 sequence (rIAB5), downstream IAB5 (dIAB5), and randomized downstream IAB5 sequence (rdIAB5) over the 99.5 percentile score (see Materials and Methods for a detailed description of how the 99.5 percentile was calculated); chi-square values obtained when comparing the number of TFBSs above the 99.5 percentile from IAB5 to rIAB5, dIAB5 to rdIAB5, and IAB5 to dIAB5; and the number of high-scoring binding sites found in the IAB5 and dIAB5 sequence (see Materials and Methods for a detailed description of high-scoring binding site). Values highlighted in orange refer to statistically significant values (p<0.05). (C) Quantitative comparison of predicted TFBSs in IAB5. Graphical representation of the number of TFBSs found in IAB5 (red), rIAB5 (black), dIAB5 (blue), and rdIAB5 (gray) for each of the transcription factors BCD, EVE, FTZ, HB, KNI and KR. The number of high-scoring TFBSs found in IAB5 (light red) and in dIAB5 (light blue) are also indicated. The general trend revealed by this analysis is that the IAB5 CRM is enriched in putative TFBSs for all the TFs analyzed (except BICOID, which is not thought to directly bind IAB5) when compared to the downstream and randomized sequences.

Detailed analysis of sequence conservation within the IAB5 enhancer CRM reveals three sub-regions that are highly conserved even in distantly related *Drosophila* species (Figure 2A). This discovery prompted us to analyze the spatial distribution of predicted TFBSs in the *D. melanogaster* IAB5 sequence to examine whether they were clustered in the regions of high conservation. In order to perform this analysis, experimentally verified TFBSs in the *D. melanogaster* genome were compiled using databases from the Eisen [39], Siggia [40] and Desplan [41] laboratories in combination with the Transfac public database [42] and additional experimentally confirmed TFBSs found in literature searches as described in the Materials and Methods section (Dataset S1). *ANN-Spec* [43] was used to align the TFBS sequences and develop an alignment matrix and a position weight matrix (PWM) for each of six TFs: BICOID (BCD), EVEN-SKIPPED (EVE), FUSHI-TARAZU (FTZ), HUNCHBACK (HB), KNIRPS (KNI) and KRUPPEL (KR) (Figure 4A) (see Materials and Methods for details). Using *Motility* [44], putative TFBSs were scored in the IAB5 enhancer CRM and the neighboring downstream IAB5 region (dIAB5) (Figure 2). The IAB5 and dIAB5 sequences were also each randomized 1000 times (rIAB5 and rdIAB5) and the 99.5 percentile score for a putative TFBS in the randomized sequence was calculated for each of the six TFs (Figure 4B). All putative TFBSs located in IAB5 and dIAB5 with scores above the 99.5 percentile score from the corresponding randomized sequence were identified (Figure 4B). Chi-square tests were used to determine if there is significant enrichment of TFBSs at IAB5 (see Materials and Methods for detailed description). In addition, a subset of TFBSs with a score above the 99.5 percentile were identified as high scoring sites by comparing the number of TFBSs predicted in the IAB5 and dIAB5 regions to the number of sites identified in the corresponding randomized sequences within the same range of scores. These computational bioinformatic approaches are described in detail in the Materials and Methods section and summarized in a concise flow chart (see Figure S9).

The IAB5 CRM sequence features significant enrichment of putative HB TFBSs when compared to both dIAB5 (p<0.001) and rIAB5 (p<0.001) (Figure 4B and 4C). There is also an enrichment of KR binding sites in IAB5 when compared to dIAB5 and rIAB5, though not statistically significant (Figure 4B and 4C). In comparison, the dIAB5 sequence is not significantly enriched in putative binding sites for any of the six TFs analyzed. Additionally, one high-scoring FTZ site, six high-scoring HB sites and one high-scoring KR site (see Materials and Methods for definition of high-scoring) were identified in IAB5 (Figure 4B and 4C and Figure S2A, S2B). These high-scoring TFBSs are not clustered in the sub-regions of the IAB5 CRM that exhibit high levels of conservation across *Drosophila* species (Figure S2A). Similar TFBS enrichment in IAB5 compared to dIAB5 was not observed for BCD or EVE. These results are in agreement with the known functional activities of HB, KR and FTZ with respect to the IAB5 CRM. HB and KR are known transcriptional repressors that act through binding IAB5, while FTZ is a known activator of IAB5 [17]. BCD and EVE were found not to be direct regulators of IAB5 in previous TF mutant studies [17], reflected in the lack of significant TFBS enrichment for these two factors in the IAB5 CRM sequence when compared to the dIAB5 sequence (Figure 4B and 4C).

Similar bioinformatic analysis was performed on the previously identified IAB2 [45], IAB7a [11], IAB7b and IAB8 [46] embryonic enhancer CRMs from the BX-C (Table S2). In general, these other IAB enhancers also exhibit greater enrichment of high-scoring putative TFBSs than neighboring regions of equal size, comparable sequence conservation and unknown function (Figure S3, S4, S5, S6). High-scoring HB and KR TFBS are found in many of the IAB enhancer CRMs, though overall enrichment, when compared to the neighboring and randomized genomic regions, is not always statistically significant (Figure S7, S8). In particular, IAB7b exhibits a similar profile of putative TFBSs to the IAB5 enhancer, featuring an enrichment of high-scoring KR, HB and FTZ binding sites (Figure S4, S7B, S8B).

### Homeotic transformation results from a point mutation in a high-scoring predicted KRUPPEL repressor binding site

**Figure 5:**
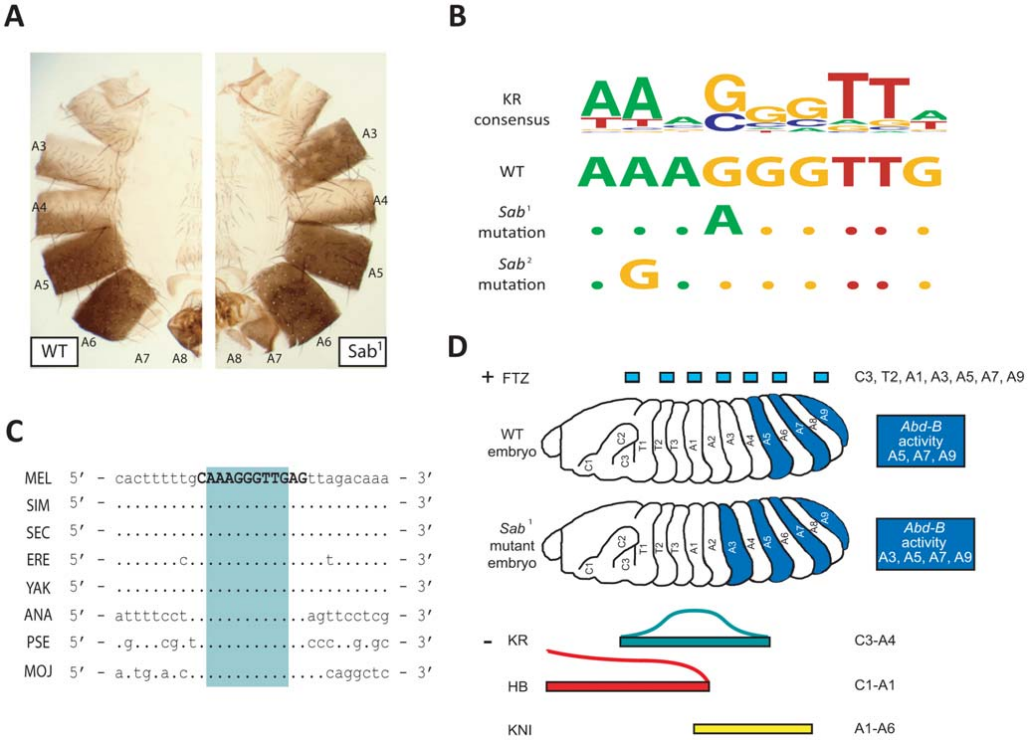
A single point mutation in a bioinformatically predicted KRUPPEL TFBS in the IAB5 enhancer CRM causes ectopic activation of *Abd-B* and a homeotic transformation in *Superabdominal* mutant embryos. (A) *Sab* homeotic mutant phenotype. In wild-type adult male flies the abdominal segments A5, A6, A7, and A8 exhibit dark pigmentation. In the *Sab^1^* mutant, abdominal segment A3, but not A4, exhibits additional dark pigmentation, suggesting a transformation towards an A5-like identity. The *Sab* ^2^ homeotic mutant exhibits a similar phenotype to *Sab* ^1^, although it has only been examined as a double mutant with *Mcp*. (B) Identification of single point mutation in the only high-scoring predicted KRUPPEL binding site in the IAB5 enhancer CRM. The IAB5 enhancer in *Sab^1^* mutants contains a single G to A substitution at base 104543 in the U31961 genomic sequence, with no additional changes in the 1027bp IAB5 region. The IAB5 enhancer in *Sab^2^* mutants contains a single A to G transition at base 104541 in the U31961 genomic sequencing, with no additional changes in the 1027 bp region. The *Sab* ^1^ and *Sab* ^2^ point mutations are in the fourth and second position, respectively, of the highest scoring predicted KRUPPEL binding site in the IAB5 enhancer. Each significantly lowers the affinity of KRUPPEL binding, as predicted by the KRUPPEL consensus binding sequence. (C) High-scoring KRUPPEL site in IAB5 is conserved across 11 Drosophila species. The IAB5 orthologous sequences from *D. melanogaster* (MEL), *D. simulans* (SIM), *D. sechellia* (SEC), *D. erecta* (ERE), *D. yakuba* (YAK), *D. ananassae* (ANA), *D. pseudoobscura* (PSE), *D. persimilis* (data not shown), *D. virilis*, (data not shown), *D. mojavensis* (MOJ) and *D. grimshawi* (data not shown) were compared by simple sequence alignment. The bioinformatically predicted high-scoring KRUPPEL binding site in the IAB5 CRM in *D. melanogaster* is 100% conserved in all these species (teal box), while the neighboring sequence does not share this high level of conservation, particularly in more distantly related species. (D) *Abdominal-B* activity in wild-type and *Sab* mutant embryos. The embryonic domains of expression for the IAB5 activator, FTZ (blue) and repressors KR (teal), KNI (yellow) and HB (red) in wild-type and *Sab* mutants are indicated. The presumptive abdominal segments in which *Abdominal-B* (*Abd-B*) is activated by the IAB5 CRM are shown in dark blue. In the wild-type embryo *Abd-B* is active in A5, A7, and A9. *Abd-B* is not active in even numbered presumptive abdominal segments (A2, A4, A6, and A8), due to the absence of the FTZ activator. In addition, IAB5-directed expression of *Abd-B* is repressed in A1, A3, and more anterior segments due to binding of the combination of the KR, KNI and HB repressor factors. In the *Sab^1^* and *Sab* ^2^ mutation, disruption of the single highest scoring KRUPPEL binding site in the IAB5 CRM prevents KRUPPEL binding and facilitates ectopic activation of *Abd-B* in A3 (see Discussion for more details).

*Superabdominal* (*Sab*) is a gain of function homeotic mutation [10]. In wild-type (WT) adult male flies, the abdominal segments A5, A6, A7 and A8 exhibit a characteristic dark pigmentation. In the *Sab^1^* mutant, abdominal segment A3, but not A4, exhibits ectopic dark pigmentation, suggesting a phenotypic transformation of A3 towards an A5-like identity (Figure 5A) [10]. Furthermore, the *Abd-B* gene is expressed in A3 of *Sab^1^* mutants, whereas it is normally repressed in this segment in WT embryos [10]. Although the molecular nature of the *Sab^1^* mutation was not known, this suggested that the IAB5 enhancer CRM may be ectopically active in the A3 segment in flies carrying the *Sab^1^* mutation (Figure 5). We hypothesized that if there is ectopic activation of IAB5, it may occur by two possible means. First, a mutation in the IAB5 sequence could create an additional activator TFBS so that IAB5 might overcome the normal repression of *Abd-B* in A3. The second possibility is that a strong repressor TFBS is mutated such that the repressor TF can no longer effectively bind and repress transcriptional activation of *Abd-B* by IAB5 in A3.

Sequencing of the *Abd-B* regulatory region of the *Sab^1^* mutant reveals a single point mutation in the center of the IAB5 CRM sequence (Figure 5B). This is the only mutation in the IAB5 CRM in *Sab^1^* mutants and this point mutation is located in the highest scoring putative KR repressor TFBS predicted in our bioinformatic analysis. The *Sab^1^* mutation presumably significantly weakens the affinity of KR for this TFBS as it substitutes the best possible base (G) at the fourth nucleotide position (base position 104543 in U31961) in the binding site to the worst possible base (A) at that position (Figure 5B). Effectively, the *Sab^1^* mutation transforms this KR TFBS from a high to very low affinity site. Furthermore, this binding site is the only statistically significant high-scoring KR site identified by our computational analysis in IAB5 and is completely conserved in *Drosophila* species from *D. melanogaster* to *D. mojavensis* (Figure 5C). The mutation of the high-scoring KR TFBS in the IAB5 enhancer CRM in *Sab^1^* flies appears to allow IAB5 to ectopically activate *Abd-B* in the A3 segment (Figure 5D). Correspondingly, *Kr* mutant embryos exhibit an anterior expansion of the *Abd-B* expression domain, which confirms our suggestion that KR is no longer acting as a repressor of the IAB5 enhancer in *Sab^1^* mutants [17]. IAB5 does not ectopically activate *Abd-B* in A4 due to the absence of the necessary FTZ activator (see Discussion for details) (Figure 5D).

**Figure 6:**
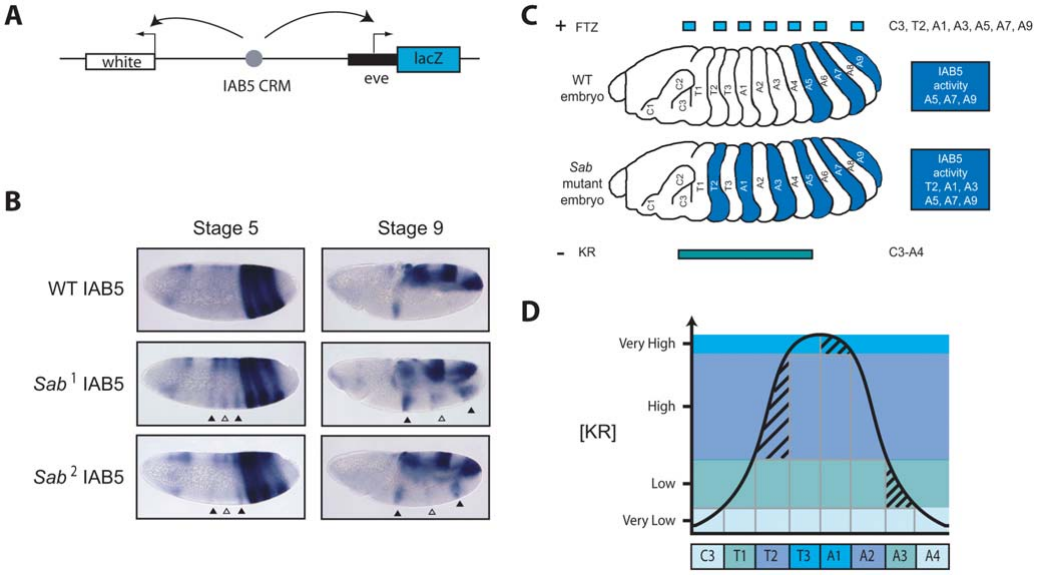
*Sab* point mutation in a bioinformatically predicted KRUPPEL TFBS in the IAB5 enhancer CRM causes anterior expansion of IAB5 in transgenic embryos. (A) *P* element construct for functional assays. An IAB5 region (gray circle) from wild-type (WT) or *Superabdominal* (*Sab* ^1^ or *Sab* ^2^) mutants is inserted between the *white* and *eve*-*lacZ* reporter genes on the *P* element reporter construct to test for WT or *Sab* IAB5 enhancer activity in transgenic *D. melanogaster* embryos. (B) Transgenic embryos carrying the *Sab* IAB5 CRMs show ectopic anterior *lacZ* expression in segments T2, A1, A3. WT IAB5 drives expression of the reporter gene *lacZ* in the presumptive fifth, seventh and ninth abdominal segments of stage 5 and stage 9 transgenic *D. melanogaster* embryos. *Sab* ^1^ and *Sab* ^2^ mutant IAB5 CRMs drive expression of the reporter gene *lacZ* not only in the presumptive fifth, seventh, and ninth abdominal segments characteristic of IAB5, but ectopic expression is also detected in three additional stripes in the presumptive third (A3) and first abdominal (A1) and second thoracic (T2) segments in transgenic *D. melanogaster* embryos (triangles). Ectopic expression of *lacZ* is consistently weaker in A1 (open triangle) compared to that in A3 and T2 segments (filled triangles). This is presumably because KRUPPEL (KR) repressor expression is highest in the A1 segment (see Discussion for more details). (C) IAB5 activity in wild-type and *Sab* mutant embryos. The embryonic domains of expression for the IAB5 activator, FTZ (blue) and repressor KR (teal) in wild-type (WT), *Sab* ^1^ *and Sab* ^2^ (Sab) mutants are indicated. The presumptive abdominal segments in which IAB5 is active are shown in dark blue. In the wild-type embryo IAB5 is active in A5, A7, and A9. IAB5 is not active in even numbered presumptive abdominal segments (A2, A4, A6, and A8), due to the absence of the FTZ activator. In addition, IAB5 is repressed in A1, A3, and more anterior segments due to binding of the combination of the KR, KNI and HB repressor factors. In either of the *Sab^1^* and *Sab* ^2^ mutations, disruption of the single highest scoring KR binding site in the IAB5 CRM alters KR binding and facilitates ectopic activation of IAB5 in A3, A1, and T2 (see Discussion for more details). (D) Model for KRUPPEL protein gradient across the presumptive anterio-posterior segments of the *Drosophila* embryo. KR repressor concentration is at its peak (very high) in segments T3 and A1 (teal). In T2 and A2 KR concentration remains high (powder blue). In T1 and A3 KR is low (light green) and in A4 and C3 (labial segment) the concentration of KR is very low (light blue). Shading indicates the specific level of KR expression in T2, A1, and A3 segments, where the sole activator of the IAB5 CRM, FTZ, is also expressed. In the *Sab* mutations, disruption of a highly conserved KR binding site in the IAB5 CRM alters KR binding and facilitates IAB5 activation in T2 and A3. IAB5-mediated activation of reporter gene expression also occurs in A1, but to a lesser extent due to the very high concentration of KR in this segment (see Discussion for detailed description).

Intriguingly, sequencing of the IAB5 region in an independently generated line with the *Sab* phenotype (*Sab^2^*) reveals a second point mutation in the exact same KR binding site as in *Sab* ^1^. This mutation is the only one in the IAB5 CRM of *Sab^2^* flies (A>G substitution at base 104541 in U31961) and would also be predicted to severely disrupt the strength of the KR binding site, based on our bioinformatic analysis (Figure 5B). To address the functional importance of the *Sab* KR site for *in vivo* repression of the IAB5 CRM, we generated transgenic *D. melanogaster* carrying a reporter construct with the IAB5 CRM harboring the *Sab^1^* or *Sab^2^* mutation (Figure 6A). In contrast to the wild-type (WT) IAB5 CRM, which drives reporter gene expression in the fifth, seventh, and ninth abdominal segments, the *Sab* mutant IAB5 CRMs drive ectopic expression in three distinct additional anterior stripes of *lacZ* (Figure 6B) and *white* (data not shown). The ectopic anterior stripes of expression driven by the *Sab* mutant IAB5 CRMs observed in Stage 5 and Stage 9 correspond to the second thoracic (T2), first (A1) and third (A3) abdominal segments and overlap with the endogenous expression pattern of the FTZ activator (Figure 6C). Additional background staining, which has been previously documented [14],[47], also appears in the anterior region in *Drosophila* embryos when using the *lacZ* ISH probe. This background expression is observed in embryos carrying a WT copy of the IAB5 enhancer and is slightly more anterior, corresponding to segment T1, than the ectopic expression seen in T2 from the *Sab* mutant IAB5 embryos (Figure 6B). The anterior expansion of IAB5 CRM activity seen in the mutant transgenic embryos confirms that the *Sab* binding site is critical for KR-mediated repression of the IAB5 enhancer CRM (see Discussion for detailed analysis).

## Discussion

### Functional evolution of CRMs across *Drosophila* species

The relative lack of overall sequence conservation at the functional CRMs of the *Abd-B* gene compared to surrounding genomic regions is consistent with emerging studies of other CRMs in *Drosophila* [16]. Indeed, only 2% of the identified conserved sequences outside of exons in mammals correspond to known CRMs [48], suggesting that sequence conservation alone may not be an indicator of regulatory function. The relative lack of information for many CRMs has in general made computational predictions of regulatory modules based on sequence conservation very challenging. Indeed, a number of other studies have suggested that the function of a CRM can be conserved in *Drosophila* [22],[49] and related insect species [20] even when the sequence varies (for a review see [50]). The results from this study indicate that the CRM sequences at the Hox genes in *Drosophila* are rapidly evolving compared to neighboring protein-coding sequences (Table S1) and therefore may be difficult to identify in other *Drosophila* species by conservation of primary sequence alone. Comparative genomic techniques based on sequence conservation to identify CRMs have been shown to be more effective in species with larger intergenic sequences, as is the case between the larger Sepsid genomes and the smaller *Drosophila* genomes [34]. Despite this fact, once a CRM from the BX-C has been identified (in this case in *D. melanogaster*), simple sequence alignment is able to identify orthologous CRMs in other *Drosophila* species with conserved functional activity. The conserved function of diverged CRMs suggests that the molecular mechanisms which regulate CRM function may also be evolutionarily conserved.

The functional conservation of orthologous CRMs in *Drosophila*, despite a lack of overall sequence conservation, has several plausible explanations. A particularly compelling argument may be that while there is an overall lack of sequence conservation in a CRM, highly conserved functional sub-regions (such as TFBSs) might be embedded within a larger region of non-functional DNA. However, previous studies have indicated that other properties of the DNA sequences in a CRM may also be conserved, such as the combinatorial architecture of TFBSs which may include features such as clustering of the binding sites [15],[16],[51]. In the context of the BX-C CRMs further bioinformatic studies, molecular analysis and transgenic assays to test the individual conserved sub-regions of the IAB5 CRM for enhancer function will clarify this issue. It will also be interesting to investigate functional compatibility in orthologous CRM sub-regions from different species. Could a functional enhancer be constructed from reciprocal halves of the IAB5 enhancer CRMs from *D. melanogaster* and *D. pseudoobscura*? Previous studies with the *eve* stripe 2 enhancer have shown that a chimeric enhancer constructed from two halves of the functional enhancers identified in *D. melanogaster* and *D. pseudoobscura* is able to recapitulate the function of the individual component enhancers [52]. Another key area for future investigation is whether the functional conservation observed for embryonic enhancers from different *Drosophila* species extends to other classes of CRMs in the BX-C and, even more broadly, to CRMs elsewhere in the genome. For example, recent evidence has indicated that some functional overlap exists between the activity of the *D. melanogaster Fab-7* and *Fab-8* insulators [53] and PREs from the BX-C [54] (Figure 1A), even in the absence of significant sequence homology. These findings suggest that orthologous insulators and PREs from different *Drosophila* species, which share a lack of underlying sequence conservation (Table S1), may also be evolutionarily conserved in function.

### Validation of computational predictions in *Hyperabdominal* homeotic mutant

*Hyperabdominal* (*Hab*) is another gain of function homeotic mutation at the BX-C [2]. The *abd-A* expression domain in *Hab* embryos is extended further anterior compared to WT embryos and the third thoracic segment (T3) is transformed toward an A2-like identity [2],[55]. The most common *Hab* phenotype is loss of the haltere and/or the third leg normally found in segment T3 and the gain of bristles which are normally found in segment A2 [2],[55]. The *Hab* mutation is a single point mutation that maps within the IAB2 embryonic enhancer sequence [55]. This single point mutation is located within the highest scoring bioinformatically predicted KR site in IAB2 in our analysis. Specifically, the mutation is a G to A substitution in the fourth base position of the KR binding site – the exact same mutation as in the highest scoring KR binding site in the IAB5 enhancer of *Sab^1^* mutants. In *Hab* embryos, mutation of the highest scoring KR binding site in IAB2 severely weakens KR binding affinity. An IAB2-directed *lacZ* reporter construct confirms that the identified single point mutation in the KR binding site of IAB2 results in ectopic gene expression in segment A3, in which KR is present [56]. The O'Connor lab also performed a DNA footprinting assay on the IAB2 enhancer CRM with KR and HB proteins [56]. These biochemical binding data offer an opportunity for us to directly test the accuracy of our computational TFBSs predictions. All the KR and HB sites identified by the DNA footprinting assay overlap with sites that we predict using bioinformatic analysis in IAB2, including the high-scoring KR binding site mutated in *Hab* flies [56].

### Molecular function of the IAB5 enhancer CRM

A critical question remains concerning the nature of the sequences which are responsible for the molecular activity of the CRM. Based on the *Sab* phenotype and the corresponding point mutation that we have characterized in the IAB5 CRM sequence in *Sab^1^* and *Sab* ^2^ mutants, we hypothesize that a single TFBS mutation can dramatically alter the functional activity of a CRM. In the case of the IAB5 transcriptional enhancer, a single G to A substitution in the fourth base position of the highest scoring computationally predicted KR binding site in the CRM, with no additional changes in the 1027bp IAB5 CRM sequence, is able to mediate ectopic activation of the enhancer and drive *Abd-B* expression in abdominal segment 3 (A3) in *Sab^1^* mutants (Figure 5). This point mutation would significantly lower the affinity of KR binding to this site, as predicted by the KR consensus binding sequence. Prior to this study, the molecular nature of the *Sab^1^* and *Sab^2^* homeotic mutations was unknown.

Our transgenic reporter gene assay reveals that the IAB5 enhancer carrying just the *Sab* ^1^ or *Sab* ^2^ single point mutation (Figure 6A) is able to ectopically activate reporter gene expression in three additional anterior segments; T2, A1 and A3 (Figure 6B). This anterior expansion of IAB5 activity corresponds precisely with the embryonic domains of KR and FTZ expression (Figure 6C). The three ectopic anterior stripes of gene expression observed therefore strongly indicate that the *Sab* point mutations leave the IAB5 CRM unable to respond to repression through KR binding. The ablation of KR binding consequently allows the IAB5 CRM to respond to a wider domain of activation by FTZ in the embryo (Figure 6C).

Given that the *Sab* IAB5 CRMs can drive ectopic gene expression in anterior segments T2, A1 and A3, an intriguing question is why in adult *Sab* mutants only A3 is transformed to an A5-like identity, while the phenotypes of the A1 and T2 segments appear unaffected. The observed differences can be resolved by considering the gradient of KR protein across the anterio-posterior axis in the early *Drosophila* embryo (Figure 6D). In A3, KR is present at a low concentration (Figure 6D) [57]. Since the mutated *Sab* KR binding site presumably has very low affinity for KR, the TF can no longer effectively bind to it in A3 and repress IAB5 activity. As a result, in A3, the *Sab* IAB5 CRM is able to direct both reporter gene expression on transgenes and *Abd-B* expression at the endogenous BX-C (Figure 6B–6D). In contrast, cells in segments T1, T3, A2 and A4 lack the presence of the known activator TF, FTZ [17], so IAB5 is inactive and *Abd-B* is not expressed (Figure 6D). In the more anterior A1 segment, KR protein concentration is at its peak (Figure 6D) [57]. Thus, at this very high concentration KR may still be able to bind (albeit in a restricted manner) to the mutated *Sab* binding site in IAB5 in the A1 segment of *Sab^1^* and *Sab* ^2^ embryos. As a result, IAB5 remains repressed and *Abd-B* is not expressed from the endogenous BX-C in A1 in these flies. In our sensitive transgenic assay we can detect ectopic reporter gene expression driven by the *Sab* IAB5 CRMs in A1 (Figure 6B). However, the expression in A1 is consistently weaker than in A3 or T2, suggesting that the *Sab* IAB5 CRM may continue to be partially repressed by KR binding in A1. It is possible that the high KR concentration in A1 ensures that despite reduced binding of KR to the IAB5 CRM at the endogenous BX-C in *Sab* mutants, it is still above a threshold level and is therefore capable of preventing activation of the *Abd-B* target gene by IAB5 (Figure 6D). Similarly, in nuclei located in segment T2 there is a high level of KR present, which may prevent activation of the *Abd-B* gene by the IAB5 CRM at the endogenous BX-C in *Sab* mutants (Figure 6D). An additional genetic component contributing to the repression of *Abd-B* in A1 and T2 in *Sab* mutants may be the high level of ULTRABITHORAX (in A1) and ANTENNAPEDIA (in T2) Hox proteins. It is feasible that the phenotypic identity of these segments is maintained in *Sab* mutants by high level expression of the endogenous Hox proteins, even if *Abd-B* is weakly expressed under the direction of the mutant IAB5 CRM. The absence of *Sab* IAB5 activity in segment C3 (labial segment) from both transgenes and at the endogenous locus, even in the presence of the FTZ activator, suggests that repression of the IAB5 enhancer CRM requires additional anterior repressor TFs.

### Bioinformatic dissection of CRMs

In an effort to directly compare the predictive specificity of our TF PWM with existing PWMs, we obtained KR PWMs from the Berkeley Drosophila Transcription Network Project (BDTNP) [39], from the Transfac repository [42] and from eCisAnalyst [58]. To determine the relative specificity with which the different matrices can indicate the location of functional binding sites, each PWM was individually used to scan through the *D. melanogaster* BX-C. The total number of predicted binding sites in the BX-C and the fraction of predicted KR binding sites that scored below the known *Sab* and *Hab* sites in the BX-C was counted (Table S3). This analysis was performed with a relatively stringent score threshold corresponding to ln(p) <−6.8 to accurately reflect existing bioinformatic approaches [58]. The new KR PWM developed in this study returns fewer predicted sites than the *BDTNP* and *eCis-Analyst* matrices, by approximately 10% (or 75 binding sites). This potentially reduces the false discovery rate for binding sites. The *Transfac* matrix returns slightly fewer hits across the BX-C, but performs worse than the newly developed PWM in predicting the rank of the *Sab* and, especially, *Hab* KRUPPEL binding sites. The new KR PWM therefore offers an improvement over the existing PWMs as it increases the stringency of prediction for functional binding sites (compared to the *Transfac* matrix), without increasing the false discovery rate (when compared to the *BDTNP* and *eCis-Analyst* matrices) (Table S3).

The agreement of our bioinformatic predictions with experimental data from the *Sab* and *Hab* homeotic mutants leads us to conclude that: (1) the position weight matrices (PWMs) for KRUPPEL and HUNCHBACK accurately predict TFBSs in CRMs; (2) the bioinformatic approach and simple statistical analysis used to obtain these results is effective; (3) the high-scoring KRUPPEL binding sites found in IAB5 and IAB2 are functional and necessary for repression of the respective CRMs; (4) KRUPPEL is a critical repressor factor, essential for establishing the correct pattern of expression of the *Abd-B* and *abd-A* Hox genes at the endogenous BX-C.

Previous studies have highlighted the functional importance of clustered binding of TFs to regulate enhancer activity [15],[16],[51]. Clustering of TFBSs has also recently been found to be a typical characteristic in blastoderm-stage *Drosophila* CRMs [39]. However, our bioinformatic analysis combined with the results in the *Hab* and *Sab* mutants suggest that clustering of KR binding sites may not be necessary for effective repression of enhancer CRM activity. This does not preclude the existence of additional KR sites within a given enhancer CRM. In some cases these additional sites may be capable of contributing to repression of CRM activity and therefore play a role in the degree of functional robustness of CRM repression. The *Hab* and *Sab* mutants also raise another intriguing question – do they represent the only two gain-of-function point mutations in the entire BX-C? The only point mutations recovered from large scale genetic screens [2] were those in the *Hab* (IAB2) and *Sab* (IAB5) KR binding sites. Intriguingly, mutations in both binding sites were recovered independently on two separate occasions, supporting the notion that the screens successfully identified all possible point mutations causing homeotic transformations of segment identity. In the case of the *Hab* and *Sab* mutations the ablation of a single KR binding site is sufficient to cause a gain-of-functional activity for the IAB2 or IAB5 embryonic enhancer CRM, respectively. Therefore, clustering analysis of TFBSs may not be sufficient to predict all functional CRMs in the genome.

It will be of interest to investigate how important clustering of putative functionally redundant TFBSs is for CRM activity. The absence of additional gain-of-function point mutations in the BX-C may indicate that at some CRMs there is extensive functional redundancy amongst clustered binding sites for critical TFs. Our bioinformatic studies to identify the sequences responsible for IAB enhancer function are therefore a critical starting point from which to perform the molecular dissection of additional CRMs active during *Drosophila* embryonic development. In particular, computational prediction of TFBSs promises to be a very useful tool to identify other sequences in the *iab* regions of the BX-C with transcriptional enhancer function. Experimental verification of the functional activity of TFBSs in conserved vs. non-conserved sub-regions of the CRMs from the BX-C and other genomic loci will greatly enhance our understanding of how evolution acts on the functional constraints of regulatory modules at the sequence level.

## Materials and Methods

### Genomic sequences

Genomic regions from the *Abd-B* gene in the *Drosophila melanogaster* bithorax complex from the annotated U31961 Genbank sequence were identified in the Berkeley Drosophila Genome Project *D. melanogaster* genome (annotated April 2004 release) and shown as ‘MEL Chr3R’ in Table S1. The class A *Abd-B* transcript and *cis*-regulatory modules from *D. melanogaster* used in the sequence conservation analysis were as described in Table S1 and the following publications: IAB8 and IAB7b [46], IAB7a and IAB6 [11], IAB5 [12], IAB2 [45], Fab-8 [14], Fab-7 [59],[60] and Mcp [45],[60], PTS7 [61], PTS6 [47], PTE [62],[63], iab8PRE [14]. Conservation analysis across the seven different *Drosophila* species was carried out using the following genome sequencing data: *D. simulans* (April 2005, Washington University School of Medicine in St. Louis, http://medschool.wustl.edu/), *D. erecta* (October 2004, Agencourt Bioscience Corporation), *D. yakuba* (April 2004, Washington University School of Medicine in St. Louis), *D. ananassae* (July 2004, The Institute for Genomic Research), *D. erecta* (October 2004, Agencourt Bioscience Corporation), *D. pseudoobscura* (July 2003, Human Genome Sequencing Center at Baylor College of Medicine, http://www.hgsc.bcm.tmc.edu/) and *D. virilis* (July 2004, Agencourt Bioscience Corporation) [35].

### Sequence alignments and identification of orthologous CRMs

Sequences were globally aligned with VISTA sequence alignment tools [37] and conserved regions were identified using default VISTA values. Level of conservation is indicated by color code: >90% red, 60–90% orange, 30–60% yellow, <30% green.

### *Abd-B* transcription studies

*In situ* hybridization probes to detect transcription of *Abd-B* in five different species of *Drosophila* were PCR-amplified using *D. melanogaster yw^67^* or *D. pseudoobscura* adult genomic DNA as a template. An orthologous region to the previously described Bexon region (exon 8 of the *D. melanogaster Abd-B* gene) [64] was identified in *D. pseudoobscura* using VISTA alignment [37]. The DNA regions were PCR amplified and cloned into pGEMT-Easy (Promega). PCR primer sequences were as follows:

Bexon mel s, 5′-GAACAAGAAGAACTCACAGC-3′ (53954);

Bexon mel as, 5′-TAGGCATAGGTGTAGGTGTAGG-3′ (55566);

Bexon pse s, 5′-GTCAAGAACGACACAACCATTC-3′ (Chr 2, 17752184);

Bexon pse as, 5′-GATCAAGCGGAGTCGATACAC-3′ (Chr 2, 17751140);

Sense and antisense RNA probes (relative to the direction of *Abd-B* transcription) were prepared using a digoxigenin (DIG) RNA-labeling kit (Roche, Gipf-Oberfrick, Switzerland). The expression pattern of *Abd-B* in *D. melanogaster*, *D. simulans*, *D. yakuba* and *D. erecta* was detected using the *D. melanogaster* Bexon probe. In *D. pseudoobscura*, *Abd-B* expression was detected using the species-specific *D. pseudoobscura* Bexon probe. Embryos from each of the five species were collected, fixed and hybridized with the appropriate probes as previously described [64].

### Bioinformatic analysis

Experimentally determined TFBSs from the *Drosophila* genome were compiled from existing databases in the Eisen [39], Siggia [40] and Desplan [41] laboratories in combination with the Transfac public database [42], with duplicated TFBSs removed. Literature searches identified additional experimentally determined TFBSs that were excluded from these four sources (see Dataset S1). TFBSs sourced from the experimental literature were characterized through DNase I footprinting and chromatin immunoprecipitation (ChIP) assays. These additional TFBSs were therefore added to generate a large composite database of experimentally determined TFBSs for six TF: BICOID (59 sites), EVEN-SKIPPED (25 sites), FUSHI-TARAZU (99 sites), HUNCHBACK (101 sites), KNIRPS (79 sites) and KRUPPEL (82 sites). The compiled TFBS sequences of varying lengths were input for the program *ANN-Spec* [43], which created a sequence alignment of a specified length, an alignment matrix and a position weight matrix (PWM) (Figure 4 and Dataset S1). The optimal length of each matrix was determined by the alignment and PWM score, the number of TFBSs from the compiled database used and by comparing our PWM to other PWMs. The PWMs created by the *ANN-Spec* algorithm take into account the frequency of each nucleotide at each position in the TFBS and the frequency of a given nucleotide and word in the genome [43]. Graphical representations of the TFBSs were created using Berkeley WebLogo [65]. The program *Motility* was used to identify putative TFBSs within a given sequence [44]. *Motility* inputs the PWM and sequence from *D. melanogaster* and outputs a list of putative binding sites and their associated scores and locations. The IAB5 enhancer CRM (IAB5) and the neighboring downstream genomic region of equal size (dIAB5) were run through the *Motility* program with each individual TF PWM. As an additional control, to determine the enrichment of binding sites that we would expect by chance in a sequence with the same length and GC content, the IAB5 and dIAB5 sequence were randomized 1000 times and also run through the *Motility* program for each individual TF PWM.

### Statistical analysis of Transcription Factor Binding Sites

Two different methods were used to analyze the output scores from *Motility*. One method is used to reduce false negatives—99.5 percentile analyses—and the other to reduce false positives—high-scoring sites. Using the program *R*, the 99.5 percentile score of binding sites for each TF found in the randomized sequences was recorded. It was then determined how many TFBSs in the IAB5 enhancer or dIAB5 region scored above the 99.5 percentile of each the corresponding randomized sequences: randomized IAB5 (rIAB5) and randomized downstream IAB5 (rdIAB5). Chi-square tests were used to determine if there was a significant enrichment of TFBSs in the IAB5 and dIAB5 regions as compared to each other and to the rIAB5 and rdIAB5 sequences, respectively. The computational bioinformatic approaches are summarized in a concise flow chart (Figure S9).

High-scoring TFBSs were identified by a more stringent mathematical analysis. For the rIAB5 enhancer or rdIAB5 sequences, the bin distribution of scores for putative binding sites for each of the six TFs was plotted on a histogram. The number of TFBSs in the IAB5 or dIAB5 sequence was then compared to the number of sites identified in the corresponding randomized sequences within the same range of scores. A chi square test was performed on the number of TFBSs in comparable score ranges for the randomized sequences (the expected value) and the IAB5 or dIAB5 sequence (the observed value) until the expected number of TFBSs in the randomized sequence is greater than one. Effectively, this approach identifies whether there are a significantly greater number of high-scoring TFBSs in the IAB5 enhancer CRM or dIAB5 region than what would be found on average in the randomized sequences.

### Construction of *P* element transgenes

Stocks used in the sequencing of *D. melanogaster*, *D. simulans*, *D. erecta*, *D. yakuba*, *D. ananassae*, *D. pseudoobscura*, and *D. virilis* were provided by the Tucson Stock Center (*D. melanogaster*: 14021-0231.36, *D. simulans*: 14021-0251.195, *D. erecta*: 14021-0224.01, *D. yakuba*: 14021-0261.01, *D. ananassae*: 14024-0371.13, *D. pseudoobscura*: 14011-0121.94, *D. virilis*: 15010-1051.87). The location of IAB5 and IAB8 orthologous regions from each species were identified by aligning the *D. melanogaster* genomic sequence to each of the other *Drosophila* genomes using VISTA [37]. These regions were PCR amplified from genomic DNA of each species. The PCR primers were designed to border the predicted IAB5 or IAB8 of each species and included a linker (bases A, T and a NotI restriction site) appended to the 5′ end of each upstream primer and a linker (bases A, T and an AscI restriction site) appended to the 5′ end of each downstream primer.

IAB5 Primers used:

*D. melanogaster* and *D. simulans*:

5′- ATGCGGCCGCTCCACTTCCGAACTTGGTCGAC-3′,

5′-ATGGCGCGCCCGATTCTGCTGGCCATGACCAT-3′;

*D. erecta*:

5′-ATGCGGCCGCTCCACTTCCGAACTTGGTCGAC-3′,

5′-ATGGCGCGCCCGATTCCGTTGGCCATGGCCAT-3′;

*D. yakuba*:

5′-ATGCGGCCGCTCCACTTCCGAACTTGGTCGGC-3′,

5′-ATGGCGCGCCCGATTCCGCTAGCCATGACCAT-3′;

*D. ananassae*:

5′-ATGCGGCCGCTGGAGGAAAAGCGGAAAATGCA-3′,

5′-ATGGCGCGCCCGATTACGATGGCCATGACCAT-3′;

*D. pseudoobscura*:

5′-ATGCGGCCGCTTCCATAATGAACCCCGCGGAA-3′,

5′-ATGGCGCGCCTTGTGGCCCTGACAGTGAAGAG-3′;

The neighboring 1027 bp genomic region downstream of IAB5 (relative to the *Abd-B* gene) in *D. melanogaster* (dIAB5) was also amplified using the following primers:

5′-ATGCGGCCGCGGCGTAGTAGTCGACTGACCCA-3′,

5′-ATGGCGCGCCCGATTGAATGTCGCCATTCGCT-3′.

IAB8 primers used:

IAB8 *D. melanogaster*

5′-ATGCGGCCGCATGGGTTTTATGTATTCATTGG-3′

5′- ATGGCGCGCCACAAAAGCCAAAAACGCTGCAG-3′

IAB8 *D. simulans*:

5′- ATGCGGCCGCATGGGATTTTTGTATTCATTGG-3′

5′- ATGGCGCGCCACAAAAGCCAAAAACGCTGCAG-3′

IAB8 *D. pseudoobscura*:

5′- ATGCGGCCGCATGCCTTTTATGTATTCATCGG-3′

5′- ATGGCGCGCCAATTGAAATCGGGAAAGAACTC-3′

The IAB5 and IAB8 genomic regions were inserted in the unique *NotI* and *AscI* sites of a previously constructed pEZ vector between the *white* and *eve*-*lacZ* reporter genes [62] (Figure 3A). The same IAB5 *D. melanogaster* primers were used to amplify the *Sab* ^1^ *and Sab* ^2^ mutant IAB5 CRMs from *Sab* ^1^ and *Sab* ^2^ mutant lines, respectively.

### *P* transformation assays and *in situ* hybridization

Reporter transgenes were introduced into the *Drosophila* germ-line using standard methods for *P* element mediated transformation [66]. Multiple transgenic lines were generated for each construct and at least two independent lines were analyzed by *in situ* hybridization. Embryos were collected, fixed and hybridized with digoxigenin-labeled *lacZ* or *white* probes as previously described [64].

### *Superabdominal* mutation analysis

The stock used to sequence the *D. melanogaster Sab^1^* mutation in the IAB5 genomic region was previously described [10] and provided by Bloomington Drosophila Stock Center (*D. melanogaster* stock number: 3497). The *Sab^2^* mutation was induced on an *Mcp* mutant background by Ed Lewis and has not been separated. The *Sab^2^* fly stock was provided by Ian Duncan. The *Sab^1^* mutation is a G to A transition at position 104543 and *Sab^2^* is an A to G transition at position 104541 on *D. melanogaster* chromosome 3R in the BX-C sequence (U31961).

## Supporting Information

Figure S1 Expression pattern of *Abdominal-B* is conserved in Drosophila species. *In situ* hybridization probes were used to detect expression of the *Abdominal-B* (*Abd-B*) transcript in *D. melanogaster* (MEL), *D. simulans* (SIM), *D. yakuba* (YAK), *D. erecta* (ERE), and *D. pseudoobscura* (PSE) embryos (described in detail in Materials and Methods). Columns show embryos at developmental stages morphologically approximate to stage 5 (left) and stage 9 (right) of *D. melanogaster* embryogenesis. The pattern of *Abd-B* expression in all of the species analyzed is very similar. At stage 5, expression is detected as a disjointed circumferential band in the presumptive abdominal region. Following germband elongation (stage 9), a clear and consistent band of expression can be seen in the posterior segments of the embryo.
(1.76 MB TIF)

Figure S2 Bioinformatic analysis of TFBSs in the IAB5 and dIAB5 genomic regions Transcription factor binding sites for FTZ (blue), KR (teal), KNI (yellow), EVE (purple), BCD (green), and HB (red) are shown below the DNA sequence. Regions of the sequence which are conserved between *D. melanogaster* and distantly related species as far as *D. pseudoobscura* are highlighted in gray. Putative sites with scores above the 99.5 percentile are shown next to predicted TFBS, with high-scoring sites (see Materials and Methods for descriptions) highlighted in bold. (A) Bioinformatically predicted TFBSs in the IAB5 region. (B) Bioinformatically predicted TFBSs in the dIAB5 region.
(0.05 MB DOC)

Figure S3 Bioinformatic analysis of TFBSs in the IAB8 genomic region Transcription factor binding sites for FTZ (blue), KR (teal), KNI (yellow), EVE (purple), BCD (green), and HB (red) are shown below the DNA sequence. Regions of the sequence which are conserved between *D. melanogaster* and distantly related species as far as *D. pseudoobscura* are highlighted in gray. Putative sites with scores above the 99.5 percentile are shown next to predicted TFBS, with high-scoring sites (see Materials and Methods for descriptions) highlighted in bold.
(0.04 MB DOC)

Figure S4 Bioinformatic analysis of TFBSs in the IAB7b genomic region. Transcription factor binding sites for FTZ (blue), KR (teal), KNI (yellow), EVE (purple), BCD (green), and HB (red) are shown below the DNA sequence. Regions of the sequence which are conserved between *D. melanogaster* and distantly related species as far as *D. pseudoobscura* are highlighted in gray. Putative sites with scores above the 99.5 percentile are shown next to predicted TFBS, with high-scoring sites (see Materials and Methods for descriptions) highlighted in bold.
(0.03 MB DOC)

Figure S5 Bioinformatic analysis of TFBSs in the IAB7a genomic region. Transcription factor binding sites for FTZ (blue), KR (teal), KNI (yellow), EVE (purple), BCD (green), and HB (red) are shown below the DNA sequence. Regions of the sequence which are conserved between *D. melanogaster* and distantly related species as far as *D. pseudoobscura* are highlighted in gray. Putative sites with scores above the 99.5 percentile are shown next to predicted TFBS, with high-scoring sites (see Materials and Methods for descriptions) highlighted in bold.
(0.06 MB DOC)

Figure S6 Bioinformatic analysis of TFBSs in the IAB2 genomic region. Transcription factor binding sites for FTZ (blue), KR (teal), KNI (yellow), EVE (purple), BCD (green), and HB (red) are shown below the DNA sequence. Regions of the sequence which are conserved between *D. melanogaster* and distantly related species as far as *D. pseudoobscura* are highlighted in gray. Putative sites with scores above the 99.5 percentile are shown next to predicted TFBS, with high-scoring sites (see Materials and Methods for descriptions) highlighted in bold.
(0.05 MB DOC)

Figure S7 Bioinformatic identification of high-scoring TFBSs in the BX-C enhancer CRMs Rows in the tables show each of the TFs; BCD, EVE, FTZ, HB, KNI, and KR. Values highlighted in orange refer to statistically significant values (p<0.05). (A) Identification of high-scoring TFBSs in IAB8. Columns show the number of TFBSs found in IAB8, randomized IAB8 sequence (rIAB8), upstream IAB8 (uIAB8), and randomized upstream IAB8 sequence (ruIAB8) over the 99.5 percentile score (see Materials and Methods for a detailed description of how the 99.5 percentile was calculated); chi-square values obtained when comparing the number of TFBSs above the 99.5 percentile from IAB8 to rIAB8, uIAB8 to ruIAB8, and IAB8 to uIAB5; and the number of highscoring binding sites found in the IAB8 and uIAB8 sequence (see methods for a detailed description of high-scoring binding site). (B) Identification of high-scoring TFBSs in IAB7b. Columns show the number of TFBSs found in IAB7b, randomized IAB7b sequence (rIAB7b), downstream IAB7b (dIAB7b), and randomized downstream IAB7b sequence (rdIAB7b) over the 99.5 percentile score (see Materials and Methods for a detailed description of how the 99.5 percentile was calculated); chi-square values obtained when comparing the number of TFBSs above the 99.5 percentile from IAB7b to rIAB7b, dIAB7b to rdIAB7b, and IAB7b to dIAB7b; and the number of high-scoring binding sites found in the IAB7b and dIAB7b sequence (see Materials and Methods for a detailed description of high-scoring binding site). (C) Identification of high-scoring TFBSs in IAB7a. Columns show the number of TFBSs found in IAB7a, randomized IAB7a sequence (rIAB7a), upstream IAB7a (uIAB7a), and randomized upstream IAB7a sequence (ruIAB7a) over the 99.5 percentile score (see Materials and Methods for a detailed description of how the 99.5 percentile was calculated); chi-square values obtained when comparing the number of TFBSs above the 99.5 percentile from IAB7a to rIAB7a, uIAB7a to ruIAB7a, and IAB7a to uIAB7a; and the number of high-scoring binding sites found in the IAB7a and uIAB7a sequence (see Materials and Methods for a detailed description of high-scoring binding site). (D) Identification of high-scoring TFBSs in IAB2. Columns show the number of TFBSs found in IAB2, randomized IAB2 sequence (rIAB2), upstream IAB2 (uIAB2), and randomized upstream IAB2 sequence (ruIAB2) over the 99.5 percentile score (see Materials and Methods for a detailed description of how the 99.5 percentile was calculated); chi-square values obtained when comparing the number of TFBSs above the 99.5 percentile from IAB2 to rIAB2, uIAB2 to ruIAB2, and IAB2 to uIAB2; and the number of high-scoring binding sites found in the IAB2 and uIAB2 sequence (see Materials and Methods for a detailed description of high-scoring binding site).
(0.98 MB TIF)

Figure S8 Quantitative comparison of predicted TFBSs in BX-C CRMs. (A) Putative TFBSs in IAB8. Graphical representation of the number of TFBSs found in IAB8 (red), rIAB8 (black), uIAB8 (blue), and ruIAB8 (gray) for each of the transcription factors BCD, EVE, FTZ, 38 HB, KNI, and KR. The number of high-scoring TFBSs found in IAB8 (light red) and in uIAB8 (light blue) are also indicated. (B) Putative TFBSs in IAB7b. Graphical representation of the number of TFBSs found in IAB7b (red), rIAB7b (black), dIAB7b (blue), and rdIAB7b (gray) for each of the transcription factors BCD, EVE, FTZ, HB, KNI, and KR. The number of high-scoring TFBSs found in IAB7b (light red) and in dIAB7b (light blue) are also indicated. (C) Putative TFBSs in IAB7a. Graphical representation of the number of TFBSs found in IAB7a (red), rIAB7a (black), uIAB7a (blue), and ruIAB7a (gray) for each of the transcription factors BCD, EVE, FTZ, HB, KNI, and KR. The number of high-scoring TFBSs found in IAB7a (light red) and in uIAB7a (light blue) are also indicated. (D) Putative TFBSs in IAB2. Graphical representation of the number of TFBSs found in IAB2 (red), rIAB2 (black), uIAB2 (blue), and ruIAB2 (gray) for each of the transcription factors BCD, EVE, FTZ, HB, KNI, and KR. The number of high-scoring TFBSs found in IAB2 (light red) and in uIAB2 (light blue) are also indicated.
(0.89 MB TIF)

Figure S9 Bioinformatics flow chart.
(0.02 MB PDF)

Table S1 Sequence conservation at the bithorax complex in *Drosophila* species. Coordinates of DNA regions from the bithorax complex (BX-C) in the *D. melanogaster* genome are shown. Numbers represent the location of the designated DNA regions in sequence from the BX-C (U39161) and on chromosome 3R of the *D. melanogaster* genome (MEL Chr3R). Level of conservation between sequences from *D. melanogaster* and six other *Drosophila* species is indicated by color code: >90% red, 60–90% orange, 30–60% yellow, <30% green (calculation for conservation is detailed in methods). The defined functional CRMs for the *Abd-B* gene are in general less conserved when compared to the exons from the neighboring Hox genes. Conservation analysis across the seven different *Drosophila* species was carried out using the following genome sequencing data: *D. simulans* (April 2005, Washington University School of Medicine in St. Louis), *D. erecta* (October 2004, Agencourt Bioscience Corporation), *D. yakuba* (April 2004, Washington University School of Medicine in St. Louis), D. ananassae (July 2004, The Institute for Genomic Research), *D. erecta* (October 2004, Agencourt Bioscience Corporation), *D. pseudoobscura* (July 2003, Human Genome Sequencing Center at Baylor College of Medicine) and *D. virilis* (July 2004, Agencourt Bioscience Corporation) [35].
(0.04 MB XLS)

Table S2 Genomic coordinates and sequence conservation of IAB enhancer CRMs and neighboring sequences. Rows show the IAB enhancers and neighboring upstream (u) or downstream (d) sequences of equal length. Level of conservation between sequences from *D. melanogaster* and six other *Drosophila* species is indicated by color code: >90% red, 60–90% orange, 30–60% yellow, <30% green (calculation for conservation is detailed in Materials and Methods).
(0.02 MB XLS)

Table S3 Comparison of the predictive specificity of KRUPPEL PWMs. predicted KRUPPEL binding sites in the *D. melanogaster* BX-C sequence (BX-C) and the percentile score of the KRUPPEL *Sab* and *Hab* binding sites when counted against all predicted KRUPPEL binding sites in the BX-C when the score threshold is set to ln(p) <−6.8. Rows show the results using different PWMs, the top most row represents the matrix developed in this study, the second row is the matrix from the Berkeley Drosophila Transcription Network Project (BDTNP) [40], the third row the matrix from Transfac [43] and the fourth row the matrix built into the online CRM-finding program eCisAnalyst [58].
(0.05 MB PDF)

Dataset S1 Compiled Transcription Factor Binding Sites database (TFBSs).
(0.07 MB DOC)
