# Supplementary figures and images for: Functional Evolution of cis-Regulatory Modules at a Homeotic Gene in Drosophila
Source: PLoS Genet. 2009 Nov 6;5(11):e1000709. doi: 10.1371/journal.pgen.1000709 (PMC2763271; doi:10.1371/journal.pgen.1000709)

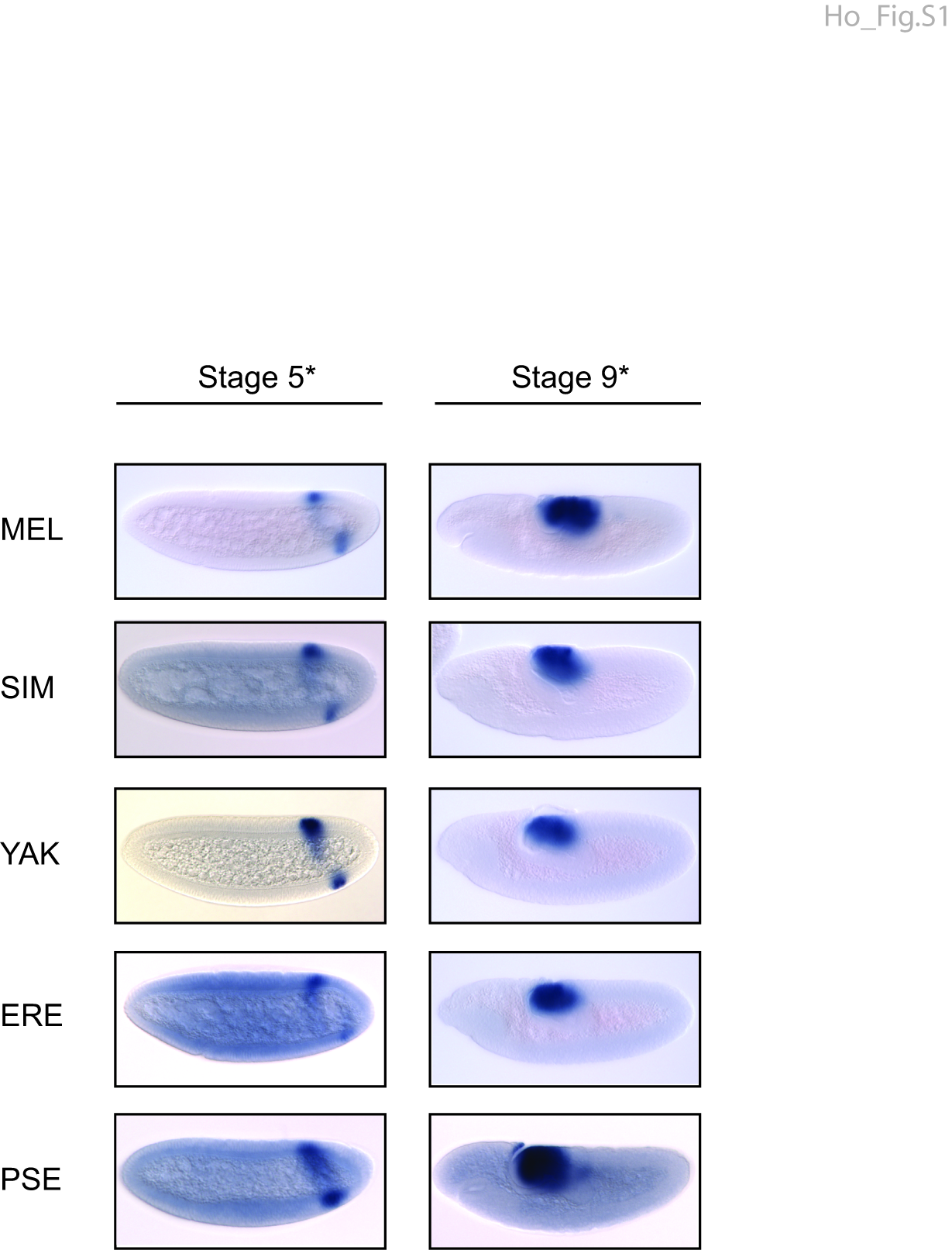

Supplement: Figure S1 — Expression pattern of Abdominal-B is conserved in Drosophila species. In situ hybridization probes were used to detect expression of the Abdominal-B (Abd-B) transcript in D. melanogaster (MEL), D. simulans (SIM), D. yakuba (YAK), D. erecta (ERE), and D. pseudoobscura (PSE) embryos (described in detail in Materials and Methods). Columns show embryos at developmental stages morphologically approximate to stage 5 (left) and stage 9 (right) of D. melanogaster embryogenesis. The pattern of Abd-B expression in all of the species analyzed is very similar. At stage 5, expression is detected as a disjointed circumferential band in the presumptive abdominal region. Following germband elongation (stage 9), a clear and consistent band of expression can be seen in the posterior segments of the embryo. (1.76 MB TIF) [file pgen.1000709.s001.tif]

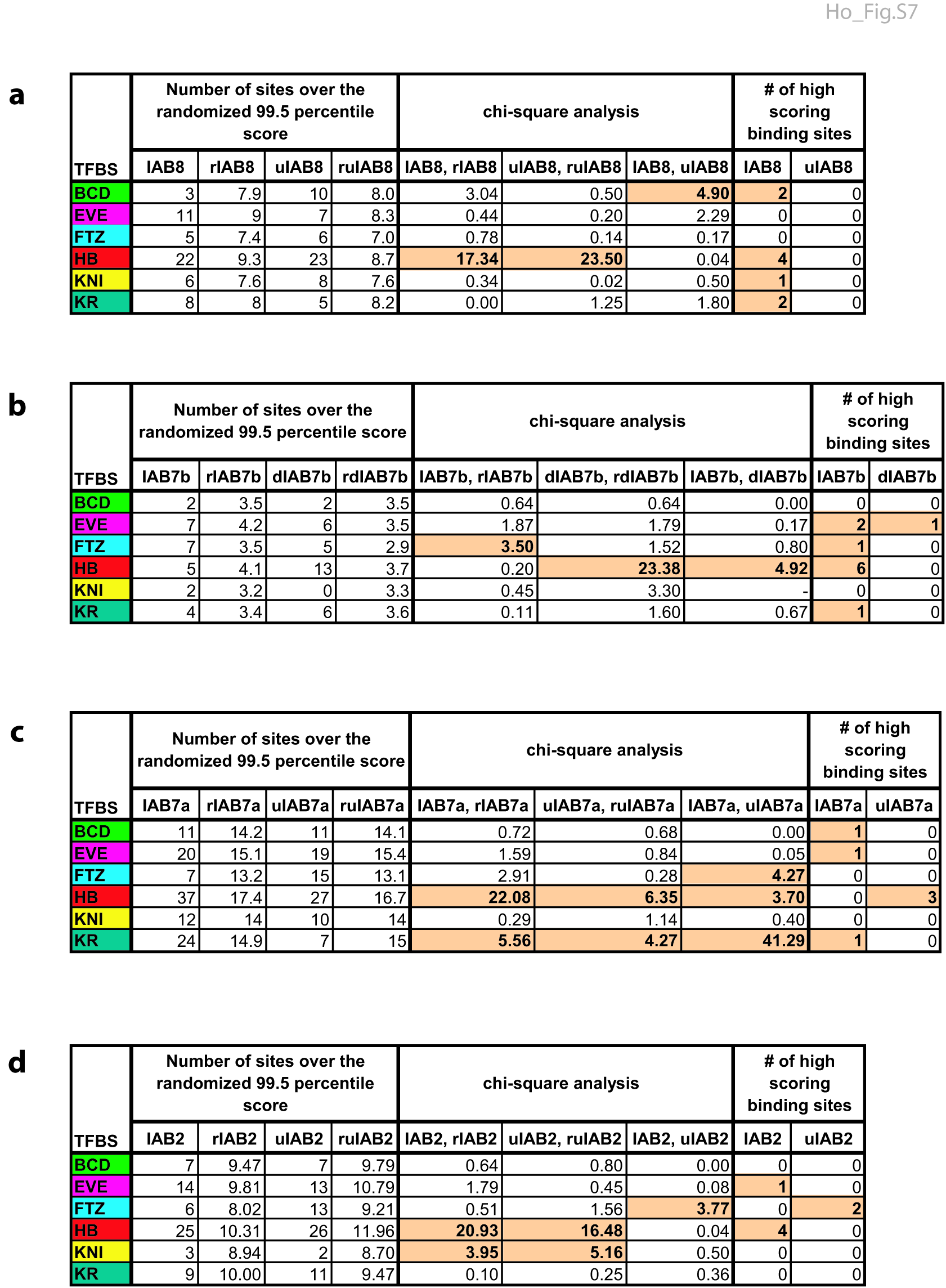

Supplement: Figure S7 — Bioinformatic identification of high-scoring TFBSs in the BX-C enhancer CRMs Rows in the tables show each of the TFs; BCD, EVE, FTZ, HB, KNI, and KR. Values highlighted in orange refer to statistically significant values (p<0.05). (A) Identification of high-scoring TFBSs in IAB8. Columns show the number of TFBSs found in IAB8, randomized IAB8 sequence (rIAB8), upstream IAB8 (uIAB8), and randomized upstream IAB8 sequence (ruIAB8) over the 99.5 percentile score (see Materials and Methods for a detailed description of how the 99.5 percentile was calculated); chi-square values obtained when comparing the number of TFBSs above the 99.5 percentile from IAB8 to rIAB8, uIAB8 to ruIAB8, and IAB8 to uIAB5; and the number of highscoring binding sites found in the IAB8 and uIAB8 sequence (see methods for a detailed description of high-scoring binding site). (B) Identification of high-scoring TFBSs in IAB7b. Columns show the number of TFBSs found in IAB7b, randomized IAB7b sequence (rIAB7b), downstream IAB7b (dIAB7b), and randomized downstream IAB7b sequence (rdIAB7b) over the 99.5 percentile score (see Materials and Methods for a detailed description of how the 99.5 percentile was calculated); chi-square values obtained when comparing the number of TFBSs above the 99.5 percentile from IAB7b to rIAB7b, dIAB7b to rdIAB7b, and IAB7b to dIAB7b; and the number of high-scoring binding sites found in the IAB7b and dIAB7b sequence (see Materials and Methods for a detailed description of high-scoring binding site). (C) Identification of high-scoring TFBSs in IAB7a. Columns show the number of TFBSs found in IAB7a, randomized IAB7a sequence (rIAB7a), upstream IAB7a (uIAB7a), and randomized upstream IAB7a sequence (ruIAB7a) over the 99.5 percentile score (see Materials and Methods for a detailed description of how the 99.5 percentile was calculated); chi-square values obtained when comparing the number of TFBSs above the 99.5 percentile from IAB7a to rIAB7a, uIAB7a to ruIAB7a, and IAB7a to [file pgen.1000709.s007.tif]

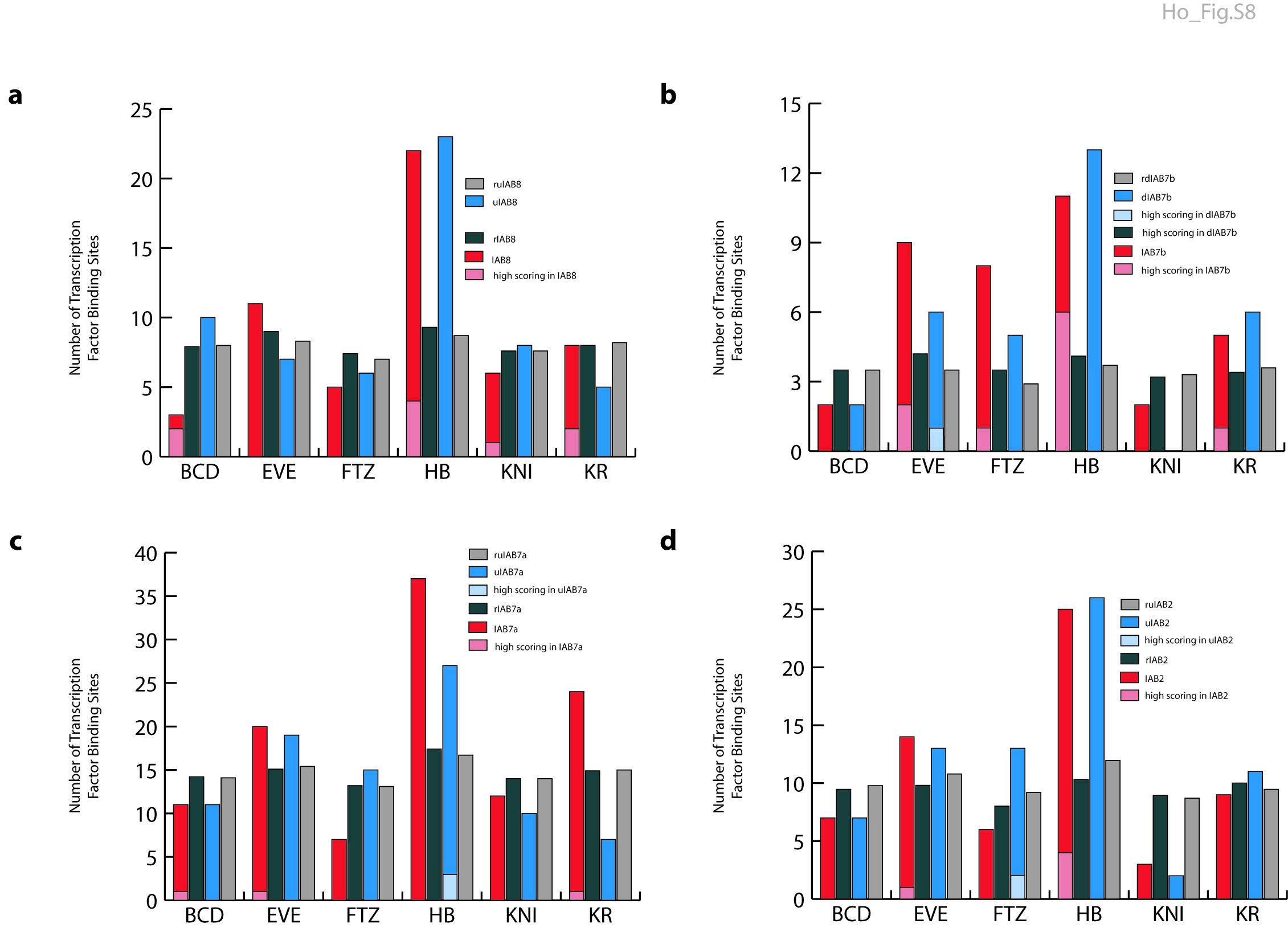

Supplement: Figure S8 — Quantitative comparison of predicted TFBSs in BX-C CRMs. (A) Putative TFBSs in IAB8. Graphical representation of the number of TFBSs found in IAB8 (red), rIAB8 (black), uIAB8 (blue), and ruIAB8 (gray) for each of the transcription factors BCD, EVE, FTZ, 38 HB, KNI, and KR. The number of high-scoring TFBSs found in IAB8 (light red) and in uIAB8 (light blue) are also indicated. (B) Putative TFBSs in IAB7b. Graphical representation of the number of TFBSs found in IAB7b (red), rIAB7b (black), dIAB7b (blue), and rdIAB7b (gray) for each of the transcription factors BCD, EVE, FTZ, HB, KNI, and KR. The number of high-scoring TFBSs found in IAB7b (light red) and in dIAB7b (light blue) are also indicated. (C) Putative TFBSs in IAB7a. Graphical representation of the number of TFBSs found in IAB7a (red), rIAB7a (black), uIAB7a (blue), and ruIAB7a (gray) for each of the transcription factors BCD, EVE, FTZ, HB, KNI, and KR. The number of high-scoring TFBSs found in IAB7a (light red) and in uIAB7a (light blue) are also indicated. (D) Putative TFBSs in IAB2. Graphical representation of the number of TFBSs found in IAB2 (red), rIAB2 (black), uIAB2 (blue), and ruIAB2 (gray) for each of the transcription factors BCD, EVE, FTZ, HB, KNI, and KR. The number of high-scoring TFBSs found in IAB2 (light red) and in uIAB2 (light blue) are also indicated. (0.89 MB TIF) [file pgen.1000709.s008.tif]

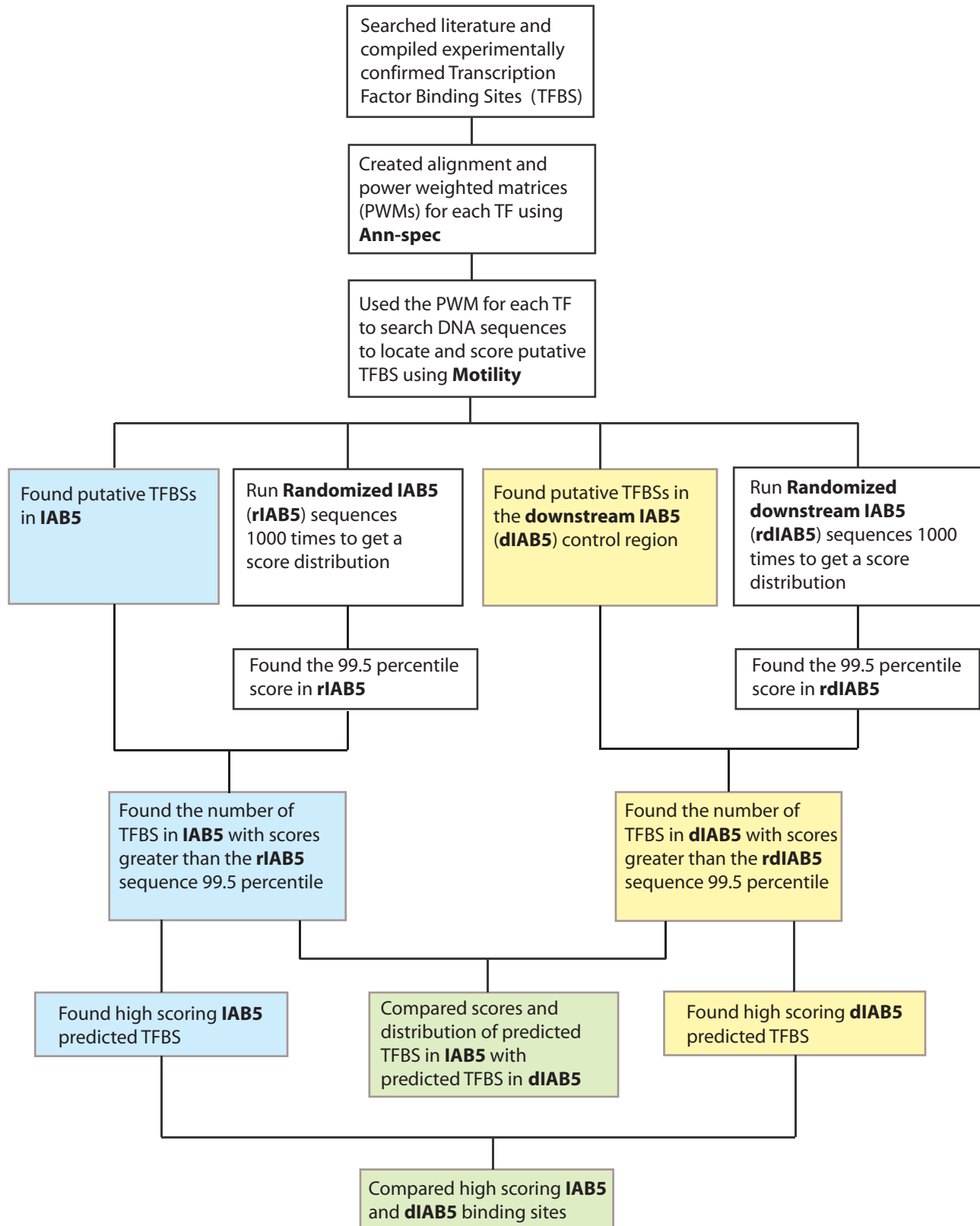

Supplement: Figure S9 — Bioinformatics flow chart. (0.02 MB PDF) [file pgen.1000709.s009.pdf]
