# Supplementary material for: Functional Evolution of cis-Regulatory Modules at a Homeotic Gene in Drosophila
Source: PLoS Genet. 2009 Nov 6;5(11):e1000709. doi: 10.1371/journal.pgen.1000709 (PMC2763271; doi:10.1371/journal.pgen.1000709)
Supplement: Figure S2 — Bioinformatic analysis of TFBSs in the IAB5 and dIAB5 genomic regions Transcription factor binding sites for FTZ (blue), KR (teal), KNI (yellow), EVE (purple), BCD (green), and HB (red) are shown below the DNA sequence. Regions of the sequence which are conserved between D. melanogaster and distantly related species as far as D. pseudoobscura are highlighted in gray. Putative sites with scores above the 99.5 percentile are shown next to predicted TFBS, with high-scoring sites (see Materials and Methods for descriptions) highlighted in bold. (A) Bioinformatically predicted TFBSs in the IAB5 region. (B) Bioinformatically predicted TFBSs in the dIAB5 region. (0.05 MB DOC) [file pgen.1000709.s002.doc]

CGATTCTGCTGGCCATGACCATGAAATGGGAGCCACAGCAGCAGCTAGGCCATAAAAGTGACATAAAATTCCCTGC

4.49 GGCCATGA ATGAAATG 10.58 **7.62** CATAAAA **7.62** CATAAAA

GACGACCTTGGGAAGCAGTAAAATTTTTAATGACTCTCACTTTCTACGGCCAACTCTCGTAGACCGTAAAACTTCGAA

10.13 CAGTAAAA TTTTAATG **11.69** 6.14 CGTAAAA

6.55 TTTTTAA

4.68 TTAATGAC

6.3 TTTAATG

CTGTGACGAGCACAGCAACACACACCATCTCACTTGAGCATCGTGGAAGAAAGAAAGCGAGAGCAGATCGCCTTGG

6.19 GATCGCCT

TTTTTTCGCCGCAGAACTGACAATGGGCCCACTGTGCGGCCATTATGTGAGGACCTCACTGGGCGCGTGTGAGTGTA

TTTTTCG 6.32 4.78 GACAATGG 4.87 GGCCATTA

TTTTTTC **6.81** 4.48 CAATGGGC

TGCGTGTGTGTCTGAATCCTTGTGGGCGGAGAACCCTTGCCTTTGTGAATCGTTTTTTATGCCCGACCTTGTTTTATG

7.79 CTGAATCC 6.36 GAACCCTTG 6.17 GAATCGTTT **7.62** TTTTATG

6.73 TTTTTTA

10.48 TTTTTATG

**7.62** TTTTATG

GCCCCGAGAGCCGCGGGCCTGGCTCACTGACATTGAACCGAACCAGAACTCAGAGCTCAGAGCTCGAGCCCATCCC

10.7 CATTGAAC 5.97 GAACTCA **6.53** CATCCC

TTTTGTCTAACTCAACCCTTTGCAAAAAGTGCGAGCTGGAGAATAACTAACGATAATTGCACGCAGGTTGCGCCTAA

TTT **8.01** CAACCCTTT CAAAAAGT 6.52 4.59 TAATTGCA

TTTAGTTTCAAGGTCCGCTGTTTTTCCGGAAATTATCATTTCATACTTTATTGCAGCAATGTGGCATAGGCAAGGCTC

4.54 GGAAATTA CATTTCAT TTTATTG 6.48

10.58

ATTTTCGTAATTGGCTCTTAAAAACTGGAAATAATTACACTCAAGGCAGGCCCAAGAAATGTGTTCGTATGCGATCG

**5.93** TAATTGGC TTAAAAA 6.55 6.7 ATGCGATC

**7.44** CTTAAAA 6.86 GATCG

CTAAGAAAAGTGAAAGGCAGCCCAGAGATCAGTAAACAAGACACTTTTTCCACATGTAAAAAGTAGTAATAAGTTA

CTA 5.98 CAGAGATC **11.2** CATGTAAA

6.63 GTAAAAA

AGATAATACTTAAAGCCAAGAGTTCCATCCCATTTTGATAAACTATACTACCACTTTTTCTATTAACGGATTCTCTCTTA

6.16 CTTAAAG AAGAGTTC 5.73 6.29 TACCACTTT 6.45 TAACGGATT

6.0 AACGGATTC

TTTACACACTTACTATATCCCACAATTTATGTTTCCCAGTCTGTCAACCTCTTATTAATTAAGCCACCCATATTTCATTA

6.16ATTTATG 7.58 ATTAAGCC 10.08 CATTA

CATCCGATTAAGCTGTCAGACACCTGTCTTCCGATTTTCCTGGCCGCCACATTGGCTTTGTGCATTTGCCGCCTCCGTC

CAT CGATTAAG 8.68

GACCAAGTTCGGAAGTGGA

ACTTCGCATCGGATTCTTTTCATGGGGCGAAACAATTTTCGATTCAATTATTCTGAATGGTTGGGAATGTGCGGAGG

10.72 TTTTCATG 5.17 TTCAATTA

TGTGAGGGAATGCGAATTCAAAATGCACACCCCGATTTAGCTGGCAGCTGAATGAAACGTGCAATCAGTGGAGGAA

TTTCCAAGCATTCCGTGCTGGTCACCATTTTTGTTAAATGCAAAGAAACTTTTGAGAGAAATTCTCCTAATCAAAATG

**11.56** GTTAAATG 6.32 TTTTGAG 8.6 CCTAATCA

6.39 ATTTTTG

GTCGTTACTCCTTTGCAGAAGGTGTGGATAGTTCAGTCCGCTGCAGTAGCGAGTGCGTTGGCCCCGATGGACCTGTC

6.26 TACTCCTTT

TGGACTTCAGTCTCCTGTCCCTGGGTAAGCTCAATTGAACTTAATTCCATCGAACCGAAACAGAAACGAATCGGGCT

7.7 GGTAAGCT 5.89 GAACTTAA 8.73 GGCT

4.58 CTCAATTG 8.73 CT

4.68 CAATTGAA

TAAGCCAAGATCCGAAGCCAAGTTGGCCAGCACCAGAGGCAGGAACGTGTTCAATTTGCGCTCATGAATGTTTCAG

TAAG GATCCGAA 5.82 6.03 AACGTGTTC TTGCGCTC 5.75

TTAAGCC

CATATAGTTATTGGATTTAGTTTCGGCACTTGCCACTAAGCATTTCTGCCATCTTCGCCTTATTCCGCTCCAAATTTTCT

8.39 GGATTTAG 6.16 TAAGCATTT

TTTTCTGCCAGCTTGCAGAGCGTGCACAAAATTCGCAAATAAGCCAAATGACGGGCGAAAACTTTCATAAGGCATCA

TTTTCTG 6.32 6.5 CACAAAA 5.02 CAAATGAC

AGTGTGCGGCTGCGATATGAGCGCCAAGCGAATATATTAAATATTTTATGAAAAGAAGAAAGTTTTTGATTTTCGTT

7.62 TTTTATG 6.76 AAAGTCTTT

ATTAAACGGAAAGTCTTTGGTAACTTGCGGACTTTGAGATGCTAAAAGTCGGTTTATCAATAAGAAAGAAATTGCTT

6.76 AAAGTCTTT 10.36 TTGAGATG

CTAAAAG 6.34

TAAGAAATGCCGTCATTCCATATCCTAAATCAAATTTAAATTAAATAATATACCGGCTAAGTCCGATGTAACTAAGCA

4.64 GCA

AATTATTATTCTAAAATGGTGAATACCATATGAGCATCTGTTTTGAGTCGCGAGTTTGTTGAACTCTGCCATCCAAAT

AATTA 10.9 CTAAAATG 6.32 TTTTGAG 5.7 GAACTCTG 4.54 CAAAT

10.22 CATCCAAA

GGCCCATCAATAAGCCGATTGAACCAATTGCCCAGGTATCTCCAAGCAAATTGGGTCAGTCGACTACTACGCC

GGC 5.34 CAATTGCC
