# Supplementary material for: Functional Evolution of cis-Regulatory Modules at a Homeotic Gene in Drosophila
Source: PLoS Genet. 2009 Nov 6;5(11):e1000709. doi: 10.1371/journal.pgen.1000709 (PMC2763271; doi:10.1371/journal.pgen.1000709)
Supplement: Figure S3 — Bioinformatic analysis of TFBSs in the IAB8 genomic region Transcription factor binding sites for FTZ (blue), KR (teal), KNI (yellow), EVE (purple), BCD (green), and HB (red) are shown below the DNA sequence. Regions of the sequence which are conserved between D. melanogaster and distantly related species as far as D. pseudoobscura are highlighted in gray. Putative sites with scores above the 99.5 percentile are shown next to predicted TFBS, with high-scoring sites (see Materials and Methods for descriptions) highlighted in bold. (0.04 MB DOC) [file pgen.1000709.s003.doc]

ATGGGTTTTATGTATTCATTGGATCTTCGATTCGAAGCGCAGGAAGTACCATATGTTGCAGTTACATCATCGCCCAAC

**7.62** TTTTATG

TCCAGCCGAGCTGCTCTAGTTTCTCCAAACCCTTTTGTTGCATCTCCGACCGGGAATTATCCACGGTCTCGAACTGTCC

**8.92** AAACCCTTT 4.96 GTCC

ATTGTTCGGACCAAAATATTTTGAGGCCCGAGCTGCTGTAAACTGTTTATGATGTGGTCATAAATGGTCCGCCTTCAC

ATTG 6.32 TTTTGAG 6.97 AAACTGTTT 6.64 TCAC

10.2 TTATGATG

GCTTTCCCTGGGTCAAAAAATTTCTACGCCTTGAAACTATGTCTACAAGCCCGTGTGCCGGATTGTCATCAGCATAAT

GCTTT **7.8** CAAAAAA **9.27** CATAAT

CCTGCCGATCTTGGCCGCATTTTGCATCACCCTCACCGCGGGCCTCTTTTCGCACTTCCATAAATCATTCCGTCCAGCA

CC

TGCCGATC 6.09

ATAGAACCCTTTCACCGATTTTTACCTGTCTGACATTCAAATGTTTTCATTCCGGAAAATGTCAGTAATATATTCATTC

GAACCCTTT **8.15** 11.33 CATTCAAA

6.63 TTTTTAC 11.06 TTCAAATG

GAGCGGGCGTCCTTTCTGGAGAAACACAAAATAGTGATTTCTTTCCAAACCAAAATAGCATTGGGAGTAAGAAAGT

6.5 CACAAAA

GCTTTAATAATACGTGACGCGTACAGAAATTAACACGTTATGCCGTTTTTATATGAAAATTGGTTAGTTATTTGTTGTT

6.8 TAACACGTT 6.55 TTTTATA

10.98 TTTATATG

AGTGACGGTTTGGATATCACGGTGCTTTGGATGCGGACAAAAATTCAATATAAAAGGCCCAAATTAAAGAATAATTC

10.22 TTTGGATG 6.39 CAAAAAT 6.55 TATAAAA

AAAGTTTTGATAGTTTTCAGCCAGAAAAGATCAATCGTTGAGATCAAGACTCAAGTGTTCAGTACGCAATAGTTTTTT

6.32 CAGAAAA **6.71** TTGAGATC 5.6 GTACGCAA **7.8**TTTTTT

GACAACATATAATAACATTGTCGGAAAATTAATTAAAAACAAACGAATTGAAAGCGTCGTTTCGTTTTCTTTCAGTAG

G 6.55 TTAAAAA

10.09 CATATAAT

GTTTCAACATAAAACGAGTTCCTATTCATTTATTTTCAAAAATATATAAATATTGGAAGCCTCCGCTTCCCTTTATGTTT

**7.62** CATAAAA 6.39 CAAAAAT 6.34 CTTTATG

TCACTTCAATTTTTCAGAAATTGTTTAACGAAATGAAAAAATTACAAATTGGGTTTACTGCTACACCATTCGGATAGC

6.81 GAAAAAA 6.18 ATTGGGTTT

CTTAGCAGTTTTAAAACATTTCGTAAGGCCCAATATGTGTATTTAAAAGGTGGTTTAGTTCTAGCTCTAGCTTATTTCT

6.37 TTTTAAA 10.4 TTTAAAAG 5.86 TTTAGTTC

6.37 TTTAAAA 6.37 TTTAAAA

ACTTACCGGAGACATAATAAGTAAGGAGCTCTTCAATTAGCAAACTTTGAGGCGGTTTCCGTTCCTCCGGTGGGCCG

10.1 CATAATAA 5.17 TTCAATTA 6.32 TTCCGTTC

4.57 CAATTAGC

AGGACTATGCTCTAGTATACATATTTTAGCAGCACCATAATTCAGGGCCGCTATTTACGTCTCGTGTGTCAGAAGCG

GAGGCCACAAATCACTTTAATTGAAGTCACGCCCCGCAAACTCAAGGGAGCCGGTCCAAAAGCTACACCACTGTCCC

10.25 CACTTTAA

10.7 TTTAATTG

5.17 TAATTGAA

TTCAGCTGCCAAACCGCAAGGCGGAAATGCTGGTAAATACATTTTTGTCCAGGCTTTTCATATCCAATATTTTTCTCAA

6.39 ATTTTTG

GTCGCACACGCGAATTTGTAATCCACATTCACAATTGGCCAACCGAACGCGCGCGGATGCGGATTCGAATGCGGAT

**9.34** TGTAATCC 5.44 CAATTGGC 5.76 GAACGCGC 7.54 GGATTCGA

10.2 TTCGAATG

GCGGACGCGGGGTCAAAGTGCTTGTTCTTTGTCATTATTATAATTAATTTTGGTATTTTTGGCAACAAACGATGCCGA

6.39 ATTTTTG

6.5 TTTTTGG

ACCGGCTCTTTTTGATTTACAGCCACTTCTTTCATTTTCTTTATATACTTTGGGTAGACTTCTCGTATACTTTTGGGTTC

6.52 CTTTTTG 6.07 TTGGGTTC

CCATCTGCAGCGTTTTTGGCTTTTGT

6.5 TTTTTGG
