# Supplementary material for: Functional Evolution of cis-Regulatory Modules at a Homeotic Gene in Drosophila
Source: PLoS Genet. 2009 Nov 6;5(11):e1000709. doi: 10.1371/journal.pgen.1000709 (PMC2763271; doi:10.1371/journal.pgen.1000709)
Supplement: Figure S4 — Bioinformatic analysis of TFBSs in the IAB7b genomic region. Transcription factor binding sites for FTZ (blue), KR (teal), KNI (yellow), EVE (purple), BCD (green), and HB (red) are shown below the DNA sequence. Regions of the sequence which are conserved between D. melanogaster and distantly related species as far as D. pseudoobscura are highlighted in gray. Putative sites with scores above the 99.5 percentile are shown next to predicted TFBS, with high-scoring sites (see Materials and Methods for descriptions) highlighted in bold. (0.03 MB DOC) [file pgen.1000709.s004.doc]

TTCTTTTGAATATCGCTTTTGGCCACTACGATCGCTGATCACCCACTTTTAGCTCGGTTCGTCTGCTATTCGGTTGTGT

6.65 TATCGCTTT 6.13 CTACGATC

6.4 GATCGCTG

TTGAAGCCATAAATTTATTTATGATGCCATTAAATCATCATTATTGTCCAGGCGACCTTGAGCCCGTAGGCTGGCGGG

10.8 CATTAAAT 4.82 TTATTGTC

GCAGAGATGTTTTTTTTTCTCACCCATTTCGAGACACATTCCCTTTCTTTTCATTTCTGTTCTCATTTCATTTCTTTTATTT

6.81 TTTTTTC 10.58 CATTTCAT

6.28 TCACCCATT

GCCTTTTTACTGTTGTCGCCTTTTAATGGTGCGTTTTCCTTTTAAGCCTTCTGCTCTGTTTGTGTTTGCCCGTCACTCTTT

TTTTTAC 6.63 11.69 TTTTAATG 7.44 TTTTAAG

8.02 TTTAAGCC

6.69 TAAGCCTTC

ATTTCTTTCTTTTTGCCCTTGCCTTAATTGGGTCAGCGATTCTGTGATTTGACTCAGCAAACGGCGAGCTAATTGCTCA

4.59 TAATTGGG 4.57 GCTAATTG

GAAGGTTAGCAAGTCGAGTTAGAAGTCTTAGATGTTTGTAAAAAACAATGTCAAGCTCTTAGGAAATATAATTTCGT

6.63 GTAAAAA

6.73 TAAAAAA

TATTTCCACAGCTGAATTTTGTTCAAAATCCAGGTAAGGTTTAGTCGTTTTTAATTGCAATTAGTATTTATGTAAATAT

7.86 CAAAATCC 6.55 TTTTTAA TGCAATTA 4.59

6.18 TAAGGTTTA 10.7 TTTAATTG

4.59 TAATTGCA

TTATAAAATTTGTACAATAAACTTGTTATATCTGATATTCTTATATTTTTGTTAAGGAATAGCCTAGCTATACAAATGT

TATAAAA 6.55

TTTTGCTAATTTCATCTTATTTCATTGACAATGATATTATTATTAGTCCAAATAACAACTTATGTTTTCTTGGAATTCGT

**6.03** TCATTGAC

**5.27** GACAATGA

CTTCTAGCTACCTG
