# Supplementary material for: Functional Evolution of cis-Regulatory Modules at a Homeotic Gene in Drosophila
Source: PLoS Genet. 2009 Nov 6;5(11):e1000709. doi: 10.1371/journal.pgen.1000709 (PMC2763271; doi:10.1371/journal.pgen.1000709)
Supplement: Figure S5 — Bioinformatic analysis of TFBSs in the IAB7a genomic region. Transcription factor binding sites for FTZ (blue), KR (teal), KNI (yellow), EVE (purple), BCD (green), and HB (red) are shown below the DNA sequence. Regions of the sequence which are conserved between D. melanogaster and distantly related species as far as D. pseudoobscura are highlighted in gray. Putative sites with scores above the 99.5 percentile are shown next to predicted TFBS, with high-scoring sites (see Materials and Methods for descriptions) highlighted in bold. (0.06 MB DOC) [file pgen.1000709.s005.doc]

ATTGGGATCGAGATTGCCTGGATTCCTGGGCCTGCCCAGAAACCGAGTTTCAGCAGCACTAGCAAAGCCTTATTTTT

TTGGGATC 6.35 10.2 TTTT

5.94 GATCGAGA 6.48 TTT

7.39 AAAGCCTTA

ATTGGAAATCGTTTATCCCAATAGAAAATTCCCAGCAGAGACGTGCCTTTGGTTGAGAAACAGGCCATGGAGAAGT

ATTG AAATCGTTT 6.94

ATTG

GAGATACAGTGGATTTATACTCACAGTTTCACCAACTAAATAATATTTATATTTAACAATCTTGCATAAATCAAAAATT

6.21 CATAAAT

6.39 CAAAAAT

GTTATTGGAAAATGCTAAAAAATACATTAGAACTAATGAATGAAACGATTAAAATATTATATTAATACCATAAATAAT

7.62 CTAAAAA 6.94 AAACGATTA 6.21 CATAAAT

6.73 TAAAAAA 7.97 CGATTAAA

CATAAGTACGATAACTTTAACATATAATGAAAAATTAATTAGACCGAGATTAAATGAAATTATACTACACTACCGTTC

10.09 CATATAAT 8.32 AGATTAAA 6.05 TACCGTTC

10.42 ATATAATG 11.3 ATTAAATG

CGCTGCTAAATCAAATTTTGATGTATAAACCATGTCTTAAGAAATGCCGTCACAAGCGGACTTTTGTATTATTAATGA

10.58 TTTTGATG

TTTCTGGAGAAGTTTCTAAATTAATTTATAAGGAAATAACAGCTTCGGCTCTAGAAACACTGCGAAATGGTTTTGAGC

5.06 GC

6.9 AAATGGTTT

6.32 TTTTGAG

TAATTAAGTTTCCCCTCAATCCGTGCCACTGTGTAGAGCATTTAACGCCCCCAACTCGCTTTTGTTTTTGGCCTGTCAT

TAATTA 11.56 CATTTAAC 5.02 GTCAT

6.5 TTTTTGG 10.15 CAT

TTGAGTCACAAACGGCGGAATTAGTTTTCCGTTTCGTTGGCATTTCAGATGCCGGGCATTATTCCGGACCCATCCTC

TTG 10.56 CATTTCAG

TTGAG

CCTCACATCGGCATGCGTCCCCATCTACAGAAGCCCCATCCCAACGATTTCGATTCCCCGTACCCATCTTCTCATCATT

ATGCGCTTCAGAGCTTTTCGGGATTTTCTACTTCATTGCTATTATCGGAGCCCAGTGAATGGCAGTGTGTTGGATGG

7.56 GGATTTTC 6.14 ATGG

GGTTATGCCGGTCTCATCTGAAATCACACTAATTTGGCTCGGGACTAGCACCGCCCACCGCCCACTTAGGCGTGTGT

GGTTA

AGTTGTCGTTGGCCCGATTTTATGATGATGAGTTCAGAGTTCGGGCACGTTGTGCATTAATCGCATCACTTTATGTGG

7.62 TTTTATG 5.7 CAGAGTTC 7.53 ATTAATCG 6.34 CTTTATG

10.2 TTATGATG 6.0 ATGAGTTC

AGCTAGAGCTGTCCGGACACTTGGCAACCAGGTTTCCAGCTGGGCAAAGAGTTCGATCCACGGCGGATTCCCAGGG

6.39 AAAGAGTTC 7.6 GGATTCCC

5.73 AAGAGTTC

6.09 GTTCGATC

ATTCCATAATGTCCTCACATAACAGCGATAGGCCGTAAAAAAAGAGGTCAAGCCAACACGACCCTGGAACAGATTTC

6.63 GTAAAAA

6.73 TAAAAAA

AGACGAAAACGAGAGCAGTTCGATCAAATTTATTGCTGCCGACTGACCTTCGCAAACCGGCAGTCGGGATTCAGGA

6.09 GTTCGATC 7.79 GGATTCAG

6.48 TTTATTG

TTCAAATTTCATATTTCACGGCCTTAACCGGAGCGACAGGATTCCCTCGCTCGGTTCGCTCGCTCAGGGCCAATAAAA

7.6 GGATTCCC 6.05 AAA

5.1 GCCAATAA

10.2 CAATAAAA

6.48 CAATAAA

GTATTAATGACCATAACAAACGTCTTTGTGCCGCAGTTGCAGTGGTTGCCCCTCGAGGTCGTTTATCCCGCTGCGTAT

GTATTA 6.06 AACGTCTTT

TTAATGAC 4.68

TTGGTGCGCGCAACTCGTTCGGGATAACATAACAGCCCGAAACTATAAGCACTCCTATGCTGCCCGTTCTTAAGGCC

6.2 TAAGGCC

6.67 AAGGCC

TTTCTCTTACCCTTTGGTGCACAGAAAAAAAAACTTTTCTCATCTGCTATATCTTATTTTGCATTTAAAGGAGTTTTACT

TT 6.98 TTACCCTTT 6.32 CAGAAAA **12.19** CATTTAAA

TTT 6.81 GAAAAAA 10.16 TTTAAAGG

6.26 AAAGGAGTT

CGTTACTTGGAGGTCCCCTTATAAATAGAGTTTCGCAAGGTAAACAGTAACTATAGAGATAGAGTTTTGTACTGTAT

GTATCGTATTTGTAATTTAGTTTGAAATAAAATATTTGTATCCAACATATTTAAGCTAGGTTCTATTCCTAAAATTCTG

6.21 CTAAAAT

TATGCAGTTTTTTCTCCGTGTGGCCTGCTCAGTTTTCCTGTAACGTATCGAATATGTTTCTCGATTCAGTCGCTGGGGC

6.81 TTTTTTC

TCATAACGGGCCTTGAGTTATGCGAGGCTGGGGCCCGAGATAAACCCGTTCTCCCTCCCTCGCTCCCGACTTGAACT

**8.22** AAACCCGTT 6.0 GAACT

6.13 AACCCGTTC

CATTTAATCCATGAAATGGGAATGCTTTAAGTCGCTGCTCCCTGGTTAGAAATTCTATTTTATGCTTGGGTTCCCATCG

CAT 10.56 ATGAAATG 6.16 CTTTAAG 7.62 TTTTATG

CATTTAAT 11.3 6.07 TTGGGTTC

TTTAATCC 9.16

CCATCTCCATCTCCAGCTCCAGCTACATCGGCATGGCCGTTGCCATCGTCTGCCAGCGATTCAGCCAGCCAAATGGCG

4.54 CAAATGGC

ATGGCATCTTGCCATGCAATTCGAGCCGGGGGCGTGGATGTGGCCATGGATGTGGATGTGCCCGTCGGTGGGTGT

GGCCATGGAGCCCCATGGATCCTTCTTGCTCGCCTGAATCGCGCTGGGTTTCTAAGTTTTTTCGCAAACGATTTTATG

6.81 TTTTTTC 7.62 TTTTATG

6.32 TTTTTCG AAACGATTT 6.94

CATGCGCTACACCGTGCGAAGTTACATTCAGTTCTAAAAAACTTCCTACCACACATATTATGGAACATTTCCGAAGCG

5.9 TTCAGTTC

7.62 CTAAAAA

6.73 TAAAAAA

ATTGATTCCTCTGATGAAAAGAACCTAATCTCTTTCGTTCTTAAAAACTCAATACTAAAAGTAATTGCTTAAACAAATG

**9.49** CCTAATCT 7.44 CTTAAAA 6.34 CTAAAAG

6.55 TTAAAAA

6.28 TTTCGTTC

TTATCATTTAAGGGGTCCTTCTTCCCTACTGTTCGCCGAGTTAATTCCAGTGTATTTGTAATTTAAGCCAGAGAGCCAA

CATTTAAG 11.28 8.02 TTTAAGCC

6.06 TAAGGGGTC

ATGTGAAAAACTGAAAAAGGGCAAAAAATGTGATGTAAAGGAGAAAACAAGAAAAGAAAGGCGAAAAAATAAAA

7.8 CAAAAAA 6.81 GAAAAAA

GCTGCTTCTCGACGACGACATAAAACTTGCAAAATAAGTTTCGCATGTTTCAGCCAACTCAGGTAAAGAAAAAAGGG

7.62 CATAAA 6.81 GAAAAAA

GCCAAAAGCAAAAGGGCGGTCGTGGATTTGCCAAAGGCCAGATGATGAGGGGCGGCAAAGGGCTTTCGGTGCCTT

8.55 GGATTGC 7.41 AAAGGGCTT

6.99 AAGGGCTTT

GAATATGCAGCAAATATTCTTCAAACGGCTCTGACGTCGGCTCCGTTTTATATGCGCTGTGCTCAGAGGGCGGAATG

6.11 AAACGGCTC 6.55 TTTTATA

10.98 TTTATATG

GGGTCCACCCTCCTTAACAGCTCACTCCCTGGCTTTCACCTTTTTTCCCCCAACCATGCACACATCCAGGTAAATGAAA

6.81 TTTTTTC 4.74 GTAAATGA

AGATGGTCGCCGGTGAGCCAAAGGGGGCGAAACACGAAATGTACTCCAATTTAATCAAGAATTCTATTCTGTAAATT

10.32 CAATTTAA 5.12 GTAAATT

ACTTGCACAAAAACTGGAGGCAACGGAAGGTCGAGTGGGGCGAAATGTGGACTGGGAAACAGACCAACCGCCTTC

A 6.5 CACAAAA

TCTTCACAGCGCGAATTTCCATTTCATGTTACAGGAGCGTAAGAAGAAATGGAATTACCTGTAGTTGGATCCGTCAA

10.58 CATTTCAT 7.89 A

10.21 CATGTTAC

GCTTAAGTGAGAAGGGGAG

GCTTAAG
