# Supplementary material for: Functional Evolution of cis-Regulatory Modules at a Homeotic Gene in Drosophila
Source: PLoS Genet. 2009 Nov 6;5(11):e1000709. doi: 10.1371/journal.pgen.1000709 (PMC2763271; doi:10.1371/journal.pgen.1000709)
Supplement: Figure S6 — Bioinformatic analysis of TFBSs in the IAB2 genomic region. Transcription factor binding sites for FTZ (blue), KR (teal), KNI (yellow), EVE (purple), BCD (green), and HB (red) are shown below the DNA sequence. Regions of the sequence which are conserved between D. melanogaster and distantly related species as far as D. pseudoobscura are highlighted in gray. Putative sites with scores above the 99.5 percentile are shown next to predicted TFBS, with high-scoring sites (see Materials and Methods for descriptions) highlighted in bold. (0.05 MB DOC) [file pgen.1000709.s006.doc]

CGAAAATGGTACTTTGGCCAGCTGCAACACATGTCAGGCGTCGACACATGATTTGTGCTGGGGTATGGGGGTTTGG

AGGTTGGGGGCGTACATTTCTGAGCTGCACTGCGAATGTCTGACTGCAAATTACGGGAAAACAAACAGATTGTTTTA

4.64 GCAAATTA 6.45 TTTTA

ACAGAGAGAGAGAGAGAAGGGAGTGATCTGATGCTAGAGCTGACGTCAGCAGCCGGCGCCCCAGGACGTCAACA

AC 5.75 GATCTGAT

CCCCGGGGTCCATTGCGGACCTGATTCAACTGCGGATTGGCATCGCACGCAGTGCGTGAAAGGGTGAAGCTACCAA

4.96 GTCCATTG 7.55 GGATTGGC 7.43 AAAGGGTGA

TACCGACACCGACTCGCCCAGCGGCAGGTCTCGCCCTGGTGGCCAGCAGCCAGCGCTTTTCCCCGATTTTCCCCGCA

TTCCTTTCTCTACTTTCTTGTCTAGTTTTTTGTTTCTTATTTTTAGCAATTTTCCATGCTACCTGTGCGAATTTTTTGCGC

**7.8** TTTTTTG 7.62 TTTTTAG **7.8** TTTTTTG

10.36 TTTCCATG

GACGGTGTGCGGAGATGCGCTCGGAATGGCACAGGCTCCGTGTTTATGTTACGGAAATCAAAAATTCGCTATCTAC

6.39 CAAAAAT

AAATTGCTGTCCCAGTCCCAGCCTAACAGCCTCTCACTTATACCCGCTTGCCGTTTTATGGCTTTCATGTCTCTTCTTTT

5.99 TACCCGCTT 7.62 TTTTATG **7.8** TTTT

6.15 TATGGCTTT

TTGCACTGCTGCCTTTGTGCCGAGTTTTCTGGCAAAAGGTTTTCATGTCAACGGCAATTTTTTATGGTTTGCAAGTTGC

TTG 6.32 TTTTCTG 10.72 TTTTCATG 4.48 GGCAATTT

6.91 AAAAGGTTT 10.48 TTTTTATG

7.06 AAAGGTTTT 6.73 TTTTTTA

10.5 CATGTCAA 7.62 TTTTATG

GACTTTCCAACGGTAACTGTTCAAGCAGACAAAATAAAAAAGGGACAATTTTTACGTAATATTATGAAAGGTGCAGA

6.73 TAAAAAA 6.63 TTTTTAC

6.14 TTTTACG

ATTGTATATGTTGCTCGGCTGCAGTTGTAAATCATTGTGATTTTTTCTAGTTTCCGCTGCATTTTTTATGAGACTGAAC

6.81 TTTTTTC 6.73 TTTTTTA

10.48 TTTTTATG

7.62 TTTTATG

AGTTGGCAAAATATTTACGGGCGAATAAGGCCGTGACATTTGAATTTACCTCAACCGATAAGAAGTATTGGTGATTC

11.06 CATTTGAA

ACTGGTGGAAAATACAAAAAACCCCAGCTCCTGTTTTTCGGATGAACGGAACAGTGATGTTAAGAAAAAAATTTATA

**7.8** CAAAAAA 6.32 TTTTTCG 6.81 GAAAAAA

6.32 GAACGGAA 10.98 TTTATA

TGTATGGACGCCGAATGGTCCCAAATGTCTTAAAATGGAAAAGAATCGTTTTATTTAATTTCATTATCCGATAATTAA

TG **11.81** TTAAAATG 6.17 GAATCGTTT 4.78 GATAATTA

7.44 CTTAAAA

ATTCAAATTAATCTTTTAAATTAATTTCTTGTTAAACAAACACTATACTTTGCTTTTAATTTAAGGAGAGCAGCAATAA

7.88 ATTAATCT 10.2 CAATAA

9.92 TTTAAATT 6.48 CAATAA

6.37 TTTTAAA 10.92 ATAA

10.4 CTTTTAAA

AATGCGAAAAACATTGTTACTGGAGTGAACAGGAAACAGTGTAAAGGGAAGGGGGCAATAGCAATTTCAATTCTAA

AA CGAAAAA 6.32 4.51 GGCAATAG 7.89 TCTAA

A

AATG

TCAACCCCCCTTTCCCGTTTTCCCCCTGCTATCCTGTGGCCAGGAATACCAAACTAATCTAAATTATTTACACGCTACA

TCA 8.34 ACTAATCT 10.04 CTACA

ATGAGCAGCTACAATTCGTCCCATAAATCACGACTCCCAGGACACACGCGCCCCTAAACAAGATTTACGACCCCCGC

ATG 6.21 CATAAAT

AATGGCCGCCAATGGCTAAGCGCAGGTGCGAGCGAGATGGCTGCTGGCTCGCAGGACGGAGTGATGGAATCCAAC

5.06 GCCAATGG

7.7 GCTAAGCG

ATGGCTGCCGCAGTGTCTGCCTTCGCACAGGTAGACACACACACACACATTGTCATAGAGAGAGGGAAAGAGCAAA

ACGTCGGCATGGCAACGCCTGAGTTTTTTCTTAGTGTGAGTGGTGAGTCGGCGCAGCCAAACCGGAATTGCGAAGA

6.81 TTTTTTC

GAGCACTCACACGTGGATGTGAATGTGGATCTGAGCGAAAAGGAGAGCGCTCACTTTGAGTCACGGCCCAACTTTG

5.72 GATCTGAG

TTATTGATTTTCCAATGCCAACTGCAGTTCCACTCATATCGAAAACTATTCGGAAAGCCTTAAATCCGCTTGGGAAAA

10.2 TTCCAATG 7.39 AAAGCCTTA

7.68 TTAAATCC

GTCTAGAGTCTAAGACGGTTTCAATTCGGGTTTCGGATTTGAGTGTTTAAGCGAGCGGCGTTGTGGGGGACTTTCGA

6.48 AGACGGTTT 8.14 GGATTTGA

GGGTTCATTGATCTTTCTAAAAATTATGAAATCTTTATTATAAGCAGAGTATGGAGGAGCTCAGTGAGAGCCAAAGG

4.57 TCATTGAT

7.62 CTAAAAA

AGCAGAAGAAGATAGGTAGAGGTAGTACAAATATTACAG
