# Supplementary material for: Functional Evolution of cis-Regulatory Modules at a Homeotic Gene in Drosophila
Source: PLoS Genet. 2009 Nov 6;5(11):e1000709. doi: 10.1371/journal.pgen.1000709 (PMC2763271; doi:10.1371/journal.pgen.1000709)
Supplement: Table S3 — Comparison of the predictive specificity of KRUPPEL PWMs. predicted KRUPPEL binding sites in the D. melanogaster BX-C sequence (BX-C) and the percentile score of the KRUPPEL Sab and Hab binding sites when counted against all predicted KRUPPEL binding sites in the BX-C when the score threshold is set to ln(p) <−6.8. Rows show the results using different PWMs, the top most row represents the matrix developed in this study, the second row is the matrix from the Berkeley Drosophila Transcription Network Project (BDTNP) [40], the third row the matrix from Transfac [43] and the fourth row the matrix built into the online CRM-finding program eCisAnalyst [58]. (0.05 MB PDF) [file pgen.1000709.s012.pdf]

KRUPPEL PWM Source	Total Sites Predicted in BX-C	Fraction of predicted KRUPPEL binding sites in BX-C with scores below those of known functional binding sites	
		Hab	*Sab*
**Present Study**	**690**	**0.884**	**0.942**
**BDTNP**	**767**	**0.970**	**0.989**
**Transfac**	**628**	**0.384**	**0.938**
**eCisAnalyst**	**756**	**0.967**	**0.931**
