# Supplementary material for: Functional Evolution of cis-Regulatory Modules at a Homeotic Gene in Drosophila
Source: PLoS Genet. 2009 Nov 6;5(11):e1000709. doi: 10.1371/journal.pgen.1000709 (PMC2763271; doi:10.1371/journal.pgen.1000709)
Supplement: Dataset S1 — Compiled Transcription Factor Binding Sites database (TFBSs). (0.07 MB DOC) [file pgen.1000709.s013.doc]

**Ho_Supplementary material**

**Transcription factor binding sites (TFBSs)**

**Bicoid**

ALR ALIGNMENT_MATRIX

ALR 1 2 3 4 5 6 7 8

ALR ------------------------------------------------

ALR A| 7 8 10 54 57 0 6 4

ALR C| 24 21 1 0 0 2 52 34

ALR G| 16 12 2 4 0 8 0 6

ALR T| 12 18 46 1 2 49 1 15

ALR CONSENSUS CCTAATCC

ALR INFORMATION 4.767 (base_e)

WMR BEST_WEIGHTS

WMR 1 2 3 4 5 6 7 8

WMR ------------------------------------------------

WMR A| -0.61 -0.54 0.03 1.71 2.00 -1.02 -0.21 -0.79

WMR C| 0.54 0.45 -0.97 -0.89 -0.81 -0.69 1.75 0.94

WMR G| 0.24 0.10 -0.57 -0.21 -0.74 0.29 -0.64 -0.25

WMR T| -0.17 -0.01 1.51 -0.61 -0.45 1.43 -0.90 0.10

ANR CONSENSUS CCTAATCC

ANR BEST_ENERGY 4.95 BEST_ITER 264

>Bicoid known binding sites (June 3, 2008)

>

>Rajewsky et. al 2002

>CT3134:tll:CG1378 8926

AAAATAAAAAACAAATATTTGCATTTC

>CT3134:tll:CG1378 8921

GCTAAGAAATGCAAATATTTGTTTTTT

>CT3134:tll:CG1378 8844

ATTAAAAACGCAATCTGAGCTCCGCA

>CT3134:tll:CG1378 8829

TGCGTTTTTAATAATAATTTTTATAAA

>CT3134:tll:CG1378 8799

TTACTTTAAAATAATTTTATTTATTAA

>CT3134:tll:CG1378 8696

AAGGCAACGCCTAATCTGGCTCAGCCG

>CT3134:tll:CG1378 8671

CCACTTGAATCCTAAAGGCTCTCAAAA

>CT3134:tll:CG1378 8644

ACAGTGCCTCTAATCTCGCTTGGTCCT

>CT3134:tll:CG1378 8596

CGATTCCTCGACTTAAGCTCGCCGCTT

>CT3134:tll:CG1378 8574

ATCTAAAATCCGTAATCTGCTTAAGCG

>CT11169:Kr:CG3340 5900

AAAATTTAATCCGTTTCTGAAGGTAGGC

>CT11169:Kr:CG3340 5930

CAGACAAATAATCCAGCCTTAAGCATGGTGAT

>CT11169:Kr:CG3340 5951

TCAAGCTTAATCACCATGCT

>CT11169:Kr:CG3340 6001

TTTTCCTTAAATCCCTCTGTT

>CT11169:Kr:CG3340 6013

CCCTCTGTTAATCTCCGGCTT

>CT11169:Kr:CG3340 6337

TAACTGAACTAAATCCGGCTTAG

>CT7758:eve:CG2328 5113

CCGTTTGCC

>CT7758:eve:CG2328 5132

TTGACTAATAATCTCGCTGAT

>CT7758:eve:CG2328 5283

CTGGACTATAATCGCACAACGA

>CT7758:eve:CG2328 5377

CCCTTGGCTAATCCCAGCAA

>CT27633:hb:CG9786 9273

GACCAACGTAATCCCCATAGAAAACCG

>CT27633:hb:CG9786 9382

TTTCTGCTCTAATCCAGAATGGATC

>CT27633:hb:CG9786 9488

TGCCCATCTAATCCCTTGACGCGTG

>CT27633:hb:CG9786 9314

GCTCGCTGCTAAGCTGGCCAT

>CT27633:hb:CG9786 9330

GCCATCCGCTAAGCTCCCGGA

>CT27633:hb:CG9786 9343

TGGATTTGGATGATCCGGGAG

>CT15191:kni:CG4717 6930

GGGTACCTAAGCCAGCGAT

>CT15191:kni:CG4717 6940

AGGTAACGAAATCGCTGGC

>CT15191:kni:CG4717 6951

CGTTACCTAATCGCGGGA

>CT15191:kni:CG4717 6961

GGTAAGCTGATCCCGCGA

>CT15191:kni:CG4717 6972

GCTTACCTAAGCTGCAGA

>CT15191:kni:CG4717 6983

CCTAGGATAATCTGCAG

>CT15191:kni:CG4717 8186

TAGAAGTTAATCAATAAGAG

>CT15191:kni:CG4717 8624

TTCCCTGTAATCAGGCTATT

>CT3134:tll:CG1378 7154

AATGAAAGGGTTAAATAGCTG

>CT3134:tll:CG1378 7205

GAGAAAAGGGTTAAGACGCCAC

>

>Papatsenko et al. 2002

>eve2 01.seq

GCCCCTAATCCCTT

>btd 01.seq

GCCACTAATCCCGA

>sal 05.seq

AATTATAATCCCTT

>hbp2 05.seq

TGTCCTAATCCAGA

>btd 02.seq

TGCGGTAATCCGAA

>btd 01.seq

CCTCGTAATCCTTT

>btd 03.seq

ATGCATAATCCACG

>btd 04.seq

CGCATTAATCCGCC

>btd 05.seq

CGGGGTAATCCTGA

>runt 02.seq

CTGCTTAATCCGGG

>runt 03.seq

CTGCTTAATCCGGG

>hbp2 04.seq

CATCCAAATCCAAG

>sal 02.seq

CGGACAAATCCTTT

>sal 03.seq

GCTGCAAATCCGAC

>sal 04.seq

TATGCAAATCCGCC

>hbp2 06.seq

ATCCGTGATCCTCG

>sal 01.seq

ATCCGTGATCCTCG

>tll 01.seq

CTATTTAACCCTTT

>tll 02.seq

CGTCTTAACCCTTT

>kr 05.seq

TATCTTAATCTCCG

>runt 01.seq

GCGGCTAATCGGCC

>

>litsearch

>Bcd1

AGGGATTAG

>

>From footprint_matrices. Pubmed IDs "9376314", "2026328", "2065664", "1348871", "9250684", "9507113", "7617036", "2911348", "8620846", "2502714", "11731230", "8443106"

TCGAAGGGATTATA

TAATTAAGCATGGC

CGGATTTGCA

CAAAGGATTTGTCC

CTCCGGCTTATCG

CAAGCCGTTTTTC

CGAGATTATT

TGGGATTAGC

CGAAGGGATTAG

CCTTCAGAAACGGATTAAATTTTTT

AAATAATCCAGCCTTAAGCATGGTGATTAAGCTTG

TGACGGATTTTCCTTAAATCCCTCTGTTAATCTCCGGCTTAGAGC

GAACTAAATCCGGCTTAGGATTCTTG

AGTCAATCTG

CAAGATTAAA

AATGATCAAC

CTTTAGCCTT

GACAAATCGC

ACTTAAGCCG

CAAGACTAAT

TTTTAAGCCT

AAAGGCTTAAA

AAAAGTCAAA

GATAATCTGCAGCTTAGGTAAGCTGATCCCGCGATTAGG

CGAAATCGCTGGCTTAGG

GTCAAGGGATTAGA

GATCGCGGATTGAGG

ATTCTGGATTAGAGC

AAAAAATTATG

GAGCTTAGCG

CAGCTTAGCA

TTCTATGGGGATTACG

ACTTGGGATTAAAGGTGATTA

GTCATCAGATTAGC

ATTTAAATCCGTTTTGA

GTGGCTTAACTTTTG

GCGAGCTTAAGTCGAG

CAAGCGAGATTAGAGG

AAAAATTATTATTAAAAACGCAATCTGAGC

**even skipped**

ALR ALIGNMENT_MATRIX

ALR 1 2 3 4 5 6 7 8

ALR ------------------------------------------------

ALR A| 5 3 8 11 16 20 1 0

ALR C| 4 2 1 2 4 2 4 4

ALR G| 5 6 4 3 1 1 0 20

ALR T| 11 14 12 9 4 2 20 1

ALR CONSENSUS TTTAAATG

ALR INFORMATION 2.627 (base_e)

WMR BEST_WEIGHTS

WMR 1 2 3 4 5 6 7 8

WMR ------------------------------------------------

WMR A| -0.39 -0.50 0.07 0.45 0.72 1.05 -0.54 -0.63

WMR C| -0.41 -0.40 -0.68 -0.41 -0.25 -0.51 -0.35 -0.19

WMR G| -0.13 -0.13 -0.27 -0.41 -0.39 -0.51 -0.78 1.77

WMR T| 0.50 0.60 0.45 -0.05 -0.49 -0.44 1.25 -0.50

ANR CONSENSUS TTTAAATG

ANR BEST_ENERGY 4.67 BEST_ITER 2

>Li et al.

>Eve

TCAATTAAATGA

>

>Fujioka et al.

>Eve-D

CAAAATATTATGGTGTGCCCCGCTGT

>Eve-P

CACAGTCAGCGCGAATTTGCTGCGGTGAGTCGATGCTGT

>

>Hoey et al.

>Eve01

TCAGCACCG

>

>Bouchard et al.

>Eve1

GCGGTGTGTC

>Eve2

TTCGGGTGTTAATGGTTTGT

>Eve3

TTTGTAATGCC

>Eve4

CCTAATCATGCGAC

>

>From Footprint_matrices. Pubmed IDs "1671662", "2895896", "1968224", "10885752", >"2569362", "10644409", "9362473"

GTCCCGCCTCGTTATCGCCGCTCAGCACCGAGAG

CTCAGCACCGCACGATTAGCACCGTT

AAAGCTAATTGTTTTAATTTAAATGAA

CTTCAATTAACCAGTTAAATGTG

CGAAATTGATTGATATTTAATTGACATTTAATTTATT

AACATTAAAATTAATAAATTATTACAAAATGACAATACATTAACGC

CAGCTTTAATCATTCCTC

CTGAAATAAATTTACTTTAATGTT

ACAAACCATTAACACCCGAA

TGTCGCATGATTAGGGGCCATTACAAA

GTTACGATTATTACTATGTTTATTGTTATTGCAATTATTATTATTATTTTTATGGTTTTAACGATTTGAACGATTATTAGCCATAGT

GGGCCAATAATAACAATAATGCCGCTGATAATGTGGATAATAAAACAA

AACGGTAACTGTTCAA

GACAAAATAAAAAAG

TACGTAATATTAT

CAGTTGTAAATCATTGT

CGTCCTTAATTGCCTGATGAGCCATGAAATGATGTCA

**Fushi Tarazu**

ALR ALIGNMENT_MATRIX

ALR 1 2 3 4 5 6 7 8

ALR ------------------------------------------------

ALR A| 12 19 10 72 75 8 16 59

ALR C| 16 16 43 19 7 0 4 6

ALR G| 41 21 4 4 0 10 21 27

ALR T| 30 43 42 4 17 81 58 7

ALR CONSENSUS GTCAATTA

ALR INFORMATION 2.538 (base_e)

WMR BEST_WEIGHTS

WMR 1 2 3 4 5 6 7 8

WMR ------------------------------------------------

WMR A| -0.36 -0.40 -0.25 0.91 0.86 -0.38 -0.37 0.65

WMR C| -0.24 -0.12 1.04 -0.05 -0.42 -0.79 -0.54 -0.47

WMR G| 1.10 -0.22 -0.58 -0.59 -0.75 -0.23 0.08 0.16

WMR T| -0.14 0.36 0.17 -0.62 -0.04 1.03 0.46 -0.70

ANR CONSENSUS GTCAATTA

ANR BEST_ENERGY 4.60 BEST_ITER 4

>Ftz binding sites

>

>Papatsenko et. al. 2002

>ftz prox 02.seq

TGTAATTGCT

>en 01.seq

CCTAATTGTG

>en 02.seq

TTTAATTGAC

>en 05.seq

GCTAATTGGC

>en 06.seq

TTTAATGGCC

>pbx 07.seq

TTTAATGGCC

>ftz prox 03.seq

TCGAATTGTC

>ftz prox 01.seq

TCGAATTGTC

>en 03.seq

GTTAAGTGCT

>bre 41.seq

GTTAAGTGCC

>en 04.seq

ATTAATTCCT

>en 07.seq

TCTAATTAGC

>pbx 01.seq

TTTAATTTGT

>pbx 03.seq

CATAATTTTT

>pbx 02.seq

GGTAATATAG

>pbx 04.seq

GCTAATAAAA

>pbx 05.seq

GCTAATGAGC

>bre 33.seq

GTTAATGATC

>bre 20.seq

TGTGATGTGT

>bre 42.seq

TTTTATGACC

>bre 10.seq

TTTTATGTGT

>pbx 06.seq

GGTAAAAGAT

>

>litsearch

>FTZ1

GCCAAATTAGC

>FTZ2

GCTAAAGTG

>FTZ3

TATTTCATAAT

>FTZ4

TGCATTAG

>

>transfac database public

>DROME$ANTP_12

CGTATATAATATATAAGCAATTAAGGTAAACAGT

>DROME$EN_01

TAAATTAAATgTCAATTAAATaTCAATCAATT

>DROME$EN_02

ACATTTAACTGGTTAATTGA

>DROME$EVE_32

TCAGCACCGcacgaTTAGCACCG

>DROME$FTZ_09

GAAACATTACTGCCATTACAG

>DROME$FTZ_11

GTTTATGGATAGTAAATGCA

>

>Muller and Bienz 1992

> FTZ_101

TCTAAACAAATTAAATG

>FTZ_102

TCAAGGTAATAT

>FTZ_103

TGAAACAATCATAA

>FTZ_104

CCATGGCTAATAA

>FTZ_105

AGCTGCTAATGAGCGATCTTTTACC

>

>Qian et al. 1993

> FTZ_01

CGCACACATA

>FTZ_02

TATTTCTCATGTG

>FTZ_03

GACCTTGAAGGCGGCGTCAAAAAGTTAATGATCACCATCGGCTCT

>FTZ_04

ACGCCCGGCTTAACATTGCACTTTTTATGACCTCGTAAAAAAACTGCAC

>

>From Footprint_matrices. Pubmed IDs "9431813", "9043065", "3046753", "10885752", "1976571", "1982071", "8404855", "8601485", "1356761"

TGTATTTTCATAATTTTCATATTTCTCCG

CGACCATAAAA

AAGCACTTAAC

CGTCAATTAAC

GTCACATAAAG

GATTTATGACAC

CCTTCAATTAACCAGTTAAATGT

AAATTGATTGATATTTAATTGACATTTAATTTA

TGCGGTGTGTC

ACAAACCATTAACACCCGAA

TGTCGCATGATTAGGGGCCATTACAAA

GACAAAATGTCTTTTACA

CGAAACATTACTGCCATTACAG

GTCTCGCATTAGACTGATGTTCT

TGTTTATGGATAGTAAATGCA

TGGGTCCTTAAGCGTCTTAATTGCCCGCGTA

CAGCGCAATTGGTTTCCAAGGCGGAAATATTATTGGCACATCATAAAACTATAAAAC

CATTGTTAATGGACGTTATCCTTATTAGA

GTTAGGTATTAAGATCTGA

GCTTCATTATATTATAGATTA

ATTGTTATAGTGCACGGA

ACACAAGGTACAAGGT

CACATGACATTAT

CAAAAAGCCATCAA

CTAGGGGCCATAAGCAG

TACGGGGTCATCATTGCTAAAAG

GCAGGACAATTCGAATAAGT

TGACAGGAGCAATTACAG

GCAGGCAATTCAAGGA

TGACTTTGATTGGTGCC

TATATCCACCCATTGAG

TGAAGATTACTTCATTTA

TGTTTACCTTAATTGCTTATA

AAACATTTAATGGACA

GCTAATAGTAACA

TAATATACTTATGTCAA

CGATTACAATTACGATTACGGTTACGATTACGGTTATTATTATAATAATGGGTTTA

TGTGCGACTGATT

CGGTTCTAATTTTTTACGATTATTGTTATGATTATTTTT

ACGGCCTTTATGATCTTAGCGGGCTGACAATTGGGA

CTATAAAATCAATTAA

AATAATTGTACAATTTAC

CTGCTTAAGTACTTATAA

GATGTTCTTAATTAA

CAAAGCAAATT

CTATTTAATAATAA

TCACATCAG

TTGCATAATTATTTAATAATGTGAAATTAACATAATGTATTTTTGTATAATAG

TTACCATTACTC

CGACCTTGAAGGCGGCGTCAAAAAGTTAATGATCACCATCGGCTCT

ATGTTTCTCATGTG

CCGCACACATA

GCCGTCGGCCATTAAAAAAG

CGGTAAAAGATCGCTCATTAGCAGCAGCT

TTTATTAGCCATGG

ATTATGATTGTTTCA

TATATTACCTTGA

TCATTTAATTTGTTTAGA

**Hunchback**

ALR ALIGNMENT_MATRIX

ALR 1 2 3 4 5 6 7

ALR ------------------------------------------

ALR A| 1 0 1 2 25 16 17

ALR C| 1 0 2 0 1 9 11

ALR G| 2 0 1 0 3 3 71

ALR T| 97 101 97 99 72 73 2

ALR CONSENSUS TTTTTTG

ALR INFORMATION 3.411 (base_e)

WMR BEST_WEIGHTS

WMR 1 2 3 4 5 6 7

WMR ------------------------------------------

WMR A| -0.30 -0.44 -0.34 -0.11 0.56 0.09 0.01

WMR C| -0.17 -0.50 -0.46 -0.60 -0.74 -0.34 0.09

WMR G| -0.64 -0.56 -0.64 -0.50 -0.56 0.00 1.08

WMR T| 1.11 1.48 1.44 1.21 0.74 0.26 -1.18

ANR CONSENSUS TTTTTTG

ANR BEST_ENERGY 3.84 BEST_ITER 820

> 0

CTTTTTTGCATTGTTTTTACTTTGACATTTTTTGTTGTTTTTAAG

> 1

TTCTTTTTGGC

> 2

TTATTTATTTG

> 3

TTTTTTATGG

> 4

TTTTTTGTTC

> 5

TTTTTGCTGC

> 6

TTTTTTTCCC

> 7

TTTTTTATTC

> 8

ATTTTTTATGT

> 9

ATTTTTAGTG

> 10

TTTTTTG

> 11

TTTTTCTTTTTC

> 12

CTTTTTATGAT

> 13

TGTTTTTATG

> 14

TGTTTTTATGA

> 15

TTTTTTTGGC

> 16

TTTTTTTGCG

> 17

TTTTTAATGC

> 18

ATTTTTTGTGT

> 19

GTTTTTACGA

> 20

CTTTTTTGTGG

> 21

CTTTTTTGTGT

> 22

TATTTTTATGA

> 23

CTTTTTTATGG

> 24

ATTTTTTATGG

> 25

GATTTTTAGTG

> 26

CTTTTTTGTTT

> 27

CTTTTTTATGT

> 28

ATTTTTTGTTC

> 29

ATTTTTTTTCC

> 30

TGTTTTTATGA

> 31

CTTTTTTGTCA

> 32

CATTTTTAGCG

> 33

TTTTTTTCTTC

> 34

ATTTTTTATTT

> 35

CCTTTTTATGT

> 36

ATTTTTTGCGT

> 37

CTTTTTATTG

> 38

ATTGTTTTTTGGGCAA

> 39

TTTTTTATGC

> 40

TTTTTTTTTTTAGTCC

> 41

CCACCTTTTTAAGCTA

> 42

CCAATTTTTTTCCCAA

> 43

GCCGTTTTTTGGCATC

> 44

CTTCTTTGTGG

> 45

TTCCCGTTTTGCGTTT

> 46

ATAGTTGTTTAATTAT

> 47

AATTTTTGTGG

> 48

ATAATTTTTTGTTTCT

> 49

CCACCTTTTTAAGCTA

> 50

CCAATTTTTTTCCCAA

> 51

CTTTTTTGTGG

> 52

CTCTTTACGG

> 53

ATTTTTGTGC

> 54

TTTTTTATTT

> 55

CTTTTTTATGT

> 56

AATTTTTAGCT

> 57

TTCCCGTTTTGCGTTT

> 58

ATAGTTGTTTAATTAT

> 59

TTTTTTTTTTTAGTCC

> 60

ATAATTTTTTGTTTCT

> 61

TTTTTTATTG

> 62

TTCTTTGTTC

> 63

ATTGTTTTTTGGGCAA

> 64

TTTTTAATTC

> 65

ATTTTTTGTGG

> 66

TTTTTTATGC

> 67

TTTTTTATTG

> 68

TTTTTTATTC

> 69

GCCGTTTTTTGGCATC

> 70

TTTTTTATGG

> 71

AGTTTTTCGG

> 72

TTTAGGGAACCGTTTTTTATGTGTG

> 73

AGTTTTTTTACGAGGTCATAAAAAGTGCA

>

>Rajewsky et. al. 2002

>

>CT7758:eve:CG2328 2812

GATCAGTTTTTTGTTTTGGCCG

>CT7758:eve:CG2328 2832

CGACCGATTTTTGTGCCCGGTG

>CT7758:eve:CG2328 2848

CCGGTGCTCTCTTTACGGTTTATG

>CT7758:eve:CG2328 2887

CCCAGCTTCTTTGTTCCGGGCT

>CT7758:eve:CG2328 2936

TGCAGATTTTTATGGGTCCCG

>CT7758:eve:CG2328 3038

ATCACGTTTTTTGTTCCCATTG

>CT7758:eve:CG2328 3059

GTGCGCTTTTTTCGCTGCGCT

>CT7758:eve:CG2328 3076

CGCTAGTTTTTTTCCCCGAACC

>CT7758:eve:CG2328 3108

CTCTAATTTTTTAATTCTTCACG

>CT7758:eve:CG2328 3212

CTCTCGTTTTTAAGATCCGTT

>CT7758:eve:CG2328 3260

ATTCACGTTTTTACGAGCTCGT

>CT11169:Kr:CG3340 5802

CAAATTGCTTTTTATGATCATGCAT

>CT11169:Kr:CG3340 5830

CAATTTATATTTTTTTGCTTTTCCT

>CT11169:Kr:CG3340 5875

TTACACTTTTTCTTTTCTGATCCA

>CT11169:Kr:CG3340 5917

GATTAAATTTTTTCAGACAAATA

>CT11169:Kr:CG3340 6038

CGCGACGCGTTTTTTCGCGACTCC

>CT11169:Kr:CG3340 6063

CCTGCATTGTTTTTTTTTTCAGTTTCTT

>CT11169:Kr:CG3340 6281

TTTGTTGTTTTTAAGAGAAAT

>CT11169:Kr:CG3340 6291

CTTTGACATTTTTTGTTGTTTT

>CT11169:Kr:CG3340 6305

TTGCATTGTTTTTACTTTGACA

>CT11169:Kr:CG3340 6315

TACACATATTTTTTGCATTGTTTT

>CT7758:eve:CG2328 5469

ATTATGTGTTTTTATGACTTTC

>CT7758:eve:CG2328 5551

ACTGGGTTATTTTTTTGCGCCG

>CT7758:eve:CG2328 5634

ACCCCACGATTTTTTTGGCCAA

>CT27633:hb:CG9786 9365

GTGCGCATAATTTTTTGTTTCTGCTCTA

>CT27633:hb:CG9786 8881

CCCTTTTTCCCGTTTTGCGTTTTTAATA

>CT27633:hb:CG9786 5895

CTCTTTGCCGTTTTTTGGCATCTCCGCT

>CT27633:hb:CG9786 5801

TCGCCAATTGTTTTTTGGGCAACTTTAA

>CT27633:hb:CG9786 5590

CTGTATTCCACCTTTTTAAGCTAATTTCG

>CT27633:hb:CG9786 5489

TTTAGACCAATTTTTTTCCCAAGCGGAA

>CT27633:hb:CG9786 5405

TAATAGTTTTTTTTTTTAGTCCAAAATT

>CT15191:kni:CG4717 7488

TAACGAGTTTTTGTTAAGAGTA

>CT15191:kni:CG4717 7568

CTTGACTTTTTCACTCCGAAGT

>CT15191:kni:CG4717 7692

GTTCTTTCTTTTTGGCTTTGAGC

>CT15191:kni:CG4717 7724

TTAACTCTTTTTATGAATATTT

>CT15191:kni:CG4717 7833

ATTTGTATTTTATTGAGTATAA

>CT15191:kni:CG4717 8012

AAACCTATTTTTGTATGCTATT

>CT15191:kni:CG4717 8102

ATAGTATTTTTTATAACTTTTC

>CT15191:kni:CG4717 8153

ACTATCGTTTTTTTGCTATGAA

>CT15191:kni:CG4717 8309

CAAAACCTTTTTCCGCGGGAAA

>CT15191:kni:CG4717 8367

CCGCCGGTTTTTGGGTACATTT

>CT15191:kni:CG4717 8458

TGCTCATTTTTTTGGCATGGGC

>CT15191:kni:CG4717 8476

GACTTCATTTTTAACAGCTGCT

>

>Papatsenko et al. 2002

>

>iab2 03.seq

CGAATTTTTTGCGCG

>pbx 02.seq

CGAATTTTTTGCGCG

>eve3+7 01.seq

TCAGTTTTTTGTTTT

>iab2 01.seq

CTAGTTTTTTGTTTC

>kr730 09.seq

TTTTTACTTTGACAT

>pbx 04.seq

CTTTTTTAATGGCCG

>ubx 04.seq

ATTTTTTAATGTTTC

>ubx 01.seq

AGTTTTTTATGGGCC

>ubx 02.seq

TACTTTTTATGACCT

>iab2 04.seq

AATTTTTTATGGTTT

>pbx 06.seq

CATTTTTTATGGCGC

>iab2 06.seq

CATTTTTTATGAGAC

>pbx 01.seq

TCATTTTTAAGGGAA

>pbx 05.seq

GAATTTTTAAGCGGA

>sal 01.seq

GAATTTTTAAGCGGA

>ubx 03.seq

GTTTTTTTACGAGGT

>iab2 02.seq

CTTATTTTTAGCAAT

>ubx 05.seq

GTTTTTTTTAGGGAA

>pbx 03.seq

TTTTTTATTAGCCAT

>pbx 07.seq

TGGTTTTTTACCAAC

>eve3+7 09.seq

ATTTTTTAATTCACG

>iab2 05.seq

CCCTTTTTTATTTTG

>

>Hunchback from the literature

>Hb1

CGATTTTTTT

>Hb2

TTATTTTTTT

>Hb_motif

ATTTTTTT

>

>transfac database public

>

>DROME$EN_12

TTTTTCAAGG

>DROME$EVE_29

TTATTTTTTT

>DROME$EVE_31

CGATTTTTTT

>DROME$KR_19b

ATATTTTTTTGC

>DROME$KR_21

ATTTTTTCAGA

>DROME$KR_22

CGTTTTTTCGC

>DROME$KR_23

TTGTTTTTTTTTCAG

>DROME$UBX_18

ggaacgaTTTTTTAATGtttctc

**knirps**

ALR ALIGNMENT_MATRIX

ALR 1 2 3 4 5 6 7 8

ALR ------------------------------------------------

ALR A| 2 57 28 6 15 16 33 38

ALR C| 0 6 2 68 7 26 11 8

ALR G| 77 6 4 2 34 18 12 14

ALR T| 0 10 45 3 23 19 23 19

ALR CONSENSUS GATCGnAA

ALR INFORMATION 3.331 (base_e)

WMR BEST_WEIGHTS

WMR 1 2 3 4 5 6 7 8

WMR ------------------------------------------------

WMR A| -0.29 1.01 0.47 -0.39 -0.42 -0.16 0.35 0.34

WMR C| -0.70 -0.21 -0.58 1.88 -0.24 0.41 -0.16 -0.13

WMR G| 1.85 -0.56 -0.63 -0.61 0.54 -0.12 -0.27 -0.12

WMR T| -0.86 -0.24 0.75 -0.88 0.12 -0.13 0.08 -0.09

ANR CONSENSUS GATCGCAA

ANR BEST_ENERGY 3.59 BEST_ITER 554

>knirps known binding sites (June 3, 2008)

>

>Rajewsky et. al 2002

>CT7758:eve:CG2328 2797

TGCATTAGAAAACTAGATCAGTTTTT

>CT7758:eve:CG2328 2841

AACCGTAAAGAGAGCACCGGGCACAAA

>CT7758:eve:CG2328 2864

TGGGAAATGGGAACGCGGCCATAAACC

>CT7758:eve:CG2328 3022

CGTATTAGGAAAGTAGATCACGTTT

>CT7758:eve:CG2328 3067

GAAAAAAACTAGCGCAGCGAAAA

>CT11169:Kr:CG3340 6333

TGTGTAACTGAACTAAATCCGGCTTAGG

>CG6494:h:CT20229 2620

GTTGAGGATCGTAAAAAACTGC

>CG6494:h:CT20229 2639

GACGGAGGTCGTAAAACCCGTT

>CG6494:h:CT20229 2652

GGCTTAAAAAACGGACGGAGGT

>CG6494:h:CT20229 2673

CAGAGGATGGCAGAGCAGAAAG

>CG6494:h:CT20229 2693

CTGATTTGAACTGAACGGGTCA

>CG6494:h:CT20229 2708

AGAGTTGACACCGAACTGATTT

>CG6494:h:CT20229 3137

GATCATAAAGTAGATCTTAACC

>CG6494:h:CT20229 2606

TTCGACCCGGGATCGCAGTTTTTT

>CG6494:h:CT20229 2648

CTTAAAAAACGGACGGAGGTCGTA

>CG6494:h:CT20229 2725

CCCGCTATGAGAACCCGAAAGAGT

>CG6494:h:CT20229 2755

AATCCGGCTGGAACAATAAAGTTT

>CG6494:h:CT20229 2791

TAAAAAATCGCTACACACTTCCCG

>CG6494:h:CT20229 2836

AATAAAGATTGCACTTCTAAAAAA

>CG6494:h:CT20229 2864

CAAATTGTAGGAACTTAAGCGGAA

>CG6494:h:CT20229 2886

AAATTCCGAGAAACCTAATAAATT

>CG6494:h:CT20229 2915

TTTTTTGATTAGGCAAAAGGCTTA

>CG6494:h:CT20229 2980

TAAAGAAGATGTCCTTAGGAAACT

>CG6494:h:CT20229 3041

TTTTTTATGGGAACAAGACAAAAA

>CG6494:h:CT20229 3077

AGATATCACGTGGCGTGGTATCAG

>CG6494:h:CT20229 3113

GACATGTTGTGAACTTACAAAGAC

>CG6494:h:CT20229 3127

TTACAAAGACGATCATAAAGTAGA

>

>Papatsenko et. al 2002

>hairy6 03.seq

CGGAGGTCGTAA

>hairy6 19.seq

TTATGATCGTCT

>hairy6 02.seq

TGAGGATCGTAA

>hairy6 18R.seq

TTGTGAACTTAC

>hairy6 16R.seq

ATGGGAACAAGA

>hairy6 12R.seq

GTAGGAACTTAA

>hairy6 13R.seq

CGAGAAACCTAA

>eve37 01.seq

TTAGAAAACTAG

>eve37 04.seq

TTAGGAAAGTAG

>eve37 03.seq

ATGGGAACGCGG

>hairy6 01.seq

GGGGGATCGCTG

>hairy6 08R.seq

ATGAGAACCCGA

>hairy6 06R.seq

AACTGAACGGTC

>hairy6 09R.seq

GCTGGAACAATA

>kr730 01.seq

AACTGAACTAAA

>hairy6 15R.seq

CTAAGGACATCT

>

>litsearch

>KNI_1

GATCGCTGTT

>KNI_5

GCTCTGCCAT

>KNI_7

GTTCGGTGTC

>KNI_8

GATCTACTTT

>KNI_motif1

GATCGGTATT

>KNI_motif2

GATCGGTTTT

>KNI_motif3

GATCGTTATT

>KNI_motif4

GATCGTTTTT

>KNI_motif5

GTTCGGTATT

>KNI_motif6

GTTCGGTTTT

>KNI_motif7

GTTCGTTATT

>KNI_motif8

GTTCGTTTTT

>kni1

GACCGAAATA

>kni2A

GATCTCAATG

>kni2B

GATCTCGTTC

>kni3

GAGCGACCTG

>kni4A

GCTCGACATG

>kni4B

GTTCTAATTA

>kni5

AATCTGGACT

>kniCONSENSUS1

GATCGAAATG

>kniCONSENSUS3

GATCGCAATG

>kniCONSENSUS4

GATCTAAATG

>

>From Footprint_matrices. Pubmed IDs "8626035", "1348871", "8186146", "9250684", "7607084", "1687458"

GCCCGGTGCTCTCTTT

ATGGCCGCGTTCCCAT

GCTGCGCTAGTT

TGATCTCAATGTTCTGATCTCGTTC

TGCTCGACATGTTCCCATTTTTTGTTCTAATTA

AAGTAGAGCGC

GGATCGCAGTTTTTTACGATC

GTTTTACGACCTCCGTCCGTTTTT

TGCTCTGCCATCCTCTGACCCGTTCAGTTCAAATCAGTTCGGTGTC

GGACCCATTT

GAACTGGAAC

ATTATGATTGTTTCATATTT

GTTCCCAATT

AAGCTCATTT

**Kruppel**

ALR ALIGNMENT_MATRIX

ALR 1 2 3 4 5 6 7 8 9

ALR ------------------------------------------------------

ALR A| 57 61 45 1 8 10 10 5 35

ALR C| 2 5 17 25 16 18 3 6 7

ALR G| 3 5 10 54 49 52 9 11 8

ALR T| 20 11 10 2 9 2 60 60 32

ALR CONSENSUS AAAGGGTTA

ALR INFORMATION 3.547 (base_e)

WMR BEST_WEIGHTS

WMR 1 2 3 4 5 6 7 8 9

WMR ------------------------------------------------------

WMR A| 1.04 1.23 0.76 -0.87 -0.77 -0.44 -0.43 -0.66 0.42

WMR C| -0.75 -0.19 0.06 0.61 0.20 0.27 -0.46 -0.41 -0.35

WMR G| -0.45 -0.68 -0.43 1.14 0.99 1.01 -0.16 -0.21 -0.49

WMR T| 0.16 -0.36 -0.39 -0.88 -0.43 -0.85 1.05 1.28 0.42

ANR CONSENSUS AAAGGGTTA

ANR BEST_ENERGY 3.81 BEST_ITER 704

>Kruppel (June 2, 2008)

>

>Rajewsky et. al 2002

>CT7758:eve:CG2328 4976

GCGCAATATAACCCAATAATTT

>CT7758:eve:CG2328 5112

ATGGCAAACGGATTAACACGGC

>CT7758:eve:CG2328 5300

AACGAGACCGGGTTGCGAAGTC

>CT7758:eve:CG2328 5494

GATGTCGAAGGGATTAGGGGCGC

>CT7758:eve:CG2328 5545

TAGCGAACTGGGTTATTTTTTT

>CT7758:eve:CG2328 5588

GGCTCAAAACGGGTTAAGCTCGC

>CG6494:h:CT20229 5901

CTGCCTGATTCGCAAAGAGTTT

>CG6494:h:CT20229 6013

GAGTTTCCACGGATTAGACCTC

>CG6494:h:CT20229 6035

GTTCTCTCTGGGATAGAGTTTA

>CG6494:h:CT20229 6054

TAGTCTCGCGCCGAAAGAGGTT

>CG6494:h:CT20229 2633

ATCCTCAACGGGTTTTACGACC

>CG6494:h:CT20229 2661

GAGCAGAAAGGCTTAAAAAACG

>CG6494:h:CT20229 2686

GAACTGAACGGGTCAGAGGATG

>CG6494:h:CT20229 2724

GCTATGAGAACCCGAAAGAGTT

>CG6494:h:CT20229 2924

TAGGCAAAAGGCTTAAAAAACA

>CG6494:h:CT20229 2950

GCAACCTTGGTGTTAATTGAGA

>CG6494:h:CT20229 3057

GACAAAAAAGGGTTTTGCGGAG

>CT27633:hb:CG9786 9185

ATTTAGTTAAGTCCCGCAATCCTT

>CT27633:hb:CG9786 8868

GGGAAAAAGGGGCATTTACGGAAT

>CT15191:kni:CG4717 7663

CGGCATAAAAGGGTTAAACAGGTAGCTC

>CT15191:kni:CG4717 7633

GCCCATAAAAGGGTTAAGCACATC

>CT15191:kni:CG4717 7604

GTACCAAAAGGGTTGTTCAGAC

>CT15191:kni:CG4717 7800

CTTTTAAAAGGGTTACAATTAA

>

> Papatsenko et. al 2002

>hairy7 06.seq

GAGAAAGGGTTTCGG

>hairy7 04.seq

GGCAAAGGGTTTCGC

>hairy7 05.seq

ACCAAAGGGTTCGTG

>sal 05.seq

ACAAAAGGGTAATGG

>iab2 01.seq

GTGAAAGGGTGAAGC

>sal 01.seq

AGAAATGGGTGAAAT

>sal 03.seq

GCAAAAGGATGGCAC

>sal 06.seq

ATCGAAGGGATTATA

>sal 02.seq

AGCAAAGGAGTGTCA

>hairy7 01.seq

GTAGAAGGAGTGACA

>sal 04.seq

GCCAAAGGATTTGTC

>hairy7 03.seq

GGGAAAGTGTTGACA

>eve2 01.seq

ATTATTGGGTTATAT

>hairy7 02.seq

CAAGCTGAGTTAAAA

>eve2 03.seq

TCAGCGAGATTATTA

>eve2 04.seq

GTTGTGCGATTATAG

>hairy5 02.seq

TATTTAGGGTTATTT

>

>Literature

>Kr1

TAACCCGTTT

>Kr5

TTAATCCGT

>KR_4

TTCGGGTTCT

>KR_motif2

AAAGGGTTAT

>KR_motif4

AACGGGTTAT

>KR01

ACGGGGTTTA

>KR02

AAGCGGTTAT

>KR03

AAGGGGATGA

>KR04

GACGGGTTAA

>KRcore

AAGGGGTTAA

>KR05

TGCGGGACTTAA

>Kr__

AACTGGTTAA

>_KR6

ATTCGGTTAT

>

>from Footprint_matrices. Pubmed IDs "9376314", "2507923", "1683715", "1671661", "9250684", "7607084", "9507113", "7617036", "9311990", "2797150", "1687458", "10644409"

GCAGCCATTACCCTTTTGTTGGCCAAAGGATTTGT

TGAAATTTGTGCCATCCTTTT

GTTAAGCAAAGGAGTGTCATAGAAATGGGTGAAATTCT

GTTACCCGGT

GTAACTGGGACA

GTAACACGCT

TAGACGGAAT

GAGGGACCCTG

CTGGGATTAGCCAAGGGCTTGA

ATCCAATCCCGATCCCTAGCCCGATCCCAATCCCAATCCCAATCCCT

ATTAACCAGTT

TGTCACTCCTT

TTTAACTCAGC

CTTTCCCTGCC

CGAAACCCTTT

TTTAAGCCTTT

TCTGACCCGTT

CGTAAAAACTCTTTGCGAATC

CTAATCCGTGGAAACTCTAAACTCTATCCCAGAGAGAACCTCTTTCGGCGCGAG

TGTAACCCTTTTAAAAGTC

ACTTAACTCTTTTTATGAATATTTA

CCTGTTTAACCCTTTTATGCC

TGCTTAACCCTTTTATGGGC

CATTGTACCAAAAGGGTTGTTC

TAAAAGGGGACTGT

GTCTACCCTTTT

GAAAAAGGGTAAT

AAAAGGGTCAC

CAGTGCGTGAAAGGGTGAAGCTAC
